# Traumatic Brain Injury Induces cGAS Activation and Type I Interferon Signaling in Aged Mice

**DOI:** 10.3389/fimmu.2021.710608

**Published:** 2021-08-24

**Authors:** James P. Barrett, Susan M. Knoblach, Surajit Bhattacharya, Heather Gordish-Dressman, Bogdan A. Stoica, David J. Loane

**Affiliations:** ^1^Department of Anesthesiology and Shock, Trauma and Anesthesiology Research (STAR) Center, University of Maryland School of Medicine, Baltimore, MD, United States; ^2^Center for Genetic Medicine Research, Children’s Research Institute, Children’s National Health System, Washington, DC, United States; ^3^Department of Genomics and Precision Medicine, George Washington University School of Medicine and Health Sciences, Washington, DC, United States; ^4^Center for Translational Science, Children’s Research Institute, Children’s National Health System, Washington, DC, United States; ^5^Department of Pediatrics, George Washington University School of Medicine and Health Sciences, Washington, DC, United States; ^6^Veterans Affairs (VA) Maryland Health Care System, Baltimore VA Medical Center, Baltimore, MD, United States; ^7^School of Biochemistry and Immunology, Trinity Biomedical Sciences Institute, Trinity College Dublin, Dublin, Ireland

**Keywords:** type I interferons, traumatic brain injury, aging, neuroinflammation, microglia

## Abstract

Aging adversely affects inflammatory processes in the brain, which has important implications in the progression of neurodegenerative disease. Following traumatic brain injury (TBI), aged animals exhibit worsened neurological function and exacerbated microglial-associated neuroinflammation. Type I Interferons (IFN-I) contribute to the development of TBI neuropathology. Further, the Cyclic GMP-AMP Synthase (cGAS) and Stimulator of Interferon Genes (STING) pathway, a key inducer of IFN-I responses, has been implicated in neuroinflammatory activity in several age-related neurodegenerative diseases. Here, we set out to investigate the effects of TBI on cGAS/STING activation, IFN-I signaling and neuroinflammation in young and aged C57Bl/6 male mice. Using a controlled cortical impact model, we evaluated transcriptomic changes in the injured cortex at 24 hours post-injury, and confirmed activation of key neuroinflammatory pathways in biochemical studies. TBI induced changes were highly enriched for transcripts that were involved in inflammatory responses to stress and host defense. Deeper analysis revealed that TBI increased expression of IFN-I related genes (e.g. Ifnb1, Irf7, Ifi204, Isg15) and IFN-I signaling in the injured cortex of aged compared to young mice. There was also a significant age-related increase in the activation of the DNA-recognition pathway, cGAS, which is a key mechanism to propagate IFN-I responses. Finally, enhanced IFN-I signaling in the aged TBI brain was confirmed by increased phosphorylation of STAT1, an important IFN-I effector molecule. This age-related activation of cGAS and IFN-I signaling may prove to be a mechanistic link between microglial-associated neuroinflammation and neurodegeneration in the aged TBI brain.

## Introduction

Normal aging is associated with increased inflammatory activity within the central nervous system (CNS), characterized by increased levels of pro-inflammatory cytokines and evidence of enhanced microglial activation (1–3). Similar to Alzheimer’s disease (AD), advanced age alters microglial transcriptional patterns, with increased expression of genes with a damage associated microglia (DAMs) signature and loss of genes involved in homeostasis (4). Aged animals also have exaggerated responses to peripheral immune challenges, such that systemic injections of lipopolysaccharide (LPS; Toll like receptor (TLR)-4 agonist and bacterial endotoxin) or Poly I:C (TLR-3 agonist and viral mimetic) result in significantly greater pro-inflammatory cytokine expression, increased microglial activation, and sickness behavior (5–7). Our group, and others, have shown age-related differences in the response to traumatic brain injury (TBI), which is characterized by enhanced inflammatory gene expression and dysregulated microglial activation, increased tissue loss and exacerbated neurological deficits following injury (8–10). While mounting evidence indicate exaggerated post-traumatic neuroinflammatory responses with advanced age, the underlying molecular mechanisms that mediate these worsened outcomes remain unclear.

As the primary brain sentinels for innate immunity, microglia express pattern recognition receptors (PRRs) that allow them to sense invading pathogens or viral components and responds to local injury and damage associated molecular patterns (DAMPs). PRRs are classified based on their localization into TLRs, C-type lectin receptors (CLR), retinoic acid-inducible gene-I (RIG-I)-like receptors (RLR), NOD-like receptors (NLR) and cytosolic DNA sensors (CDS). TLR and RLR signaling results in production of Type I Interferons (IFN-I; IFNα and -β) and pro-inflammatory cytokines in a cell-specific manner, whereas NLR signaling leads to the production of interleukin-1 family proteins. Previous studies have demonstrated that inhibition of IFN-I results in reduced microglial-mediated neuroinflammation following TBI leading to reduced neurodegeneration and improved long-term motor and cognitive function recovery (11, 12). Significantly, IFN-I have also been implicated in the progression of age-related neuroinflammation and cognitive decline (2, 13). Inhibition of IFN-I signaling was found to reduce pro-inflammatory cytokine expression in the aged brain and concurrently increase the expression of brain-derived neurotrophic factor (BDNF) and insulin-like growth factor 1 (IGF-1) (2).

Cyclic GMP-AMP Synthase (cGAS) and Stimulator of Interferon Genes (STING) also play a role in the induction of IFN-I following CNS injury and during neurodegenerative disease (14–19). cGAS/STING signaling is involved in the host response to infection, detecting DNA and leading to induction of IFN-I and other pro-inflammatory pathways (20, 21). Disruption of cGAS/STING signaling enhances susceptibility to bacterial and viral infection (22–24). While activation of the cGAS/STING pathway has been shown to limit viral replication during CNS viral infection (25–27), chronic and dysregulated activation may be detrimental (28, 29). Prior studies have demonstrated that cGAS/STING are upregulated after acute neural injury, and selective inhibition of cGAS or STING reduces neuroinflammation and promotes functional recovery in animal models of ischemic stroke and TBI (15, 17, 24).

The beneficial effects of cGAS/STING inhibition have been attributed to reduced IFN-I production and signaling. Interferon regulatory factors (IRFs) are a family of 9 transcription factors that can propagate IFN responses and further induce IFN-I production (30–32). Several IRFs mediate pro-inflammatory activation of macrophages (32, 33). IRF7 is upregulated during CNS injury and neurodegeneration, and it is thought to be involved in the propagation of IFN-I and pro-inflammatory responses (11, 12, 34, 35). However, in agreement with the beneficial effects of IFN-I in animal models of Multiple Sclerosis, inhibition of IRF7 has also been shown to enhance protective neuroimmune responses (36). IRF1 also induces pro-inflammatory responses in brain (37, 38), and IRF1 inhibition reduces neurodegeneration and improves outcomes following ischemic stroke (37). Overall, IFN-I are implicated in acute CNS injury and age-related neurodegeneration, including chronic neurodegenerative disorders such as AD, Parkinson’s disease and amyotrophic lateral sclerosis (2, 14, 18, 19, 39, 40).

Here, we set out to determine if cGAS/STING activity and IFN-I responses were elevated in aged mice following TBI. Our analysis demonstrates that following a focal cortical injury in aged mice there is significant upregulation of cGAS and IFN-I pathways in injured tissue, suggesting their possible role in post-traumatic neuroinflammation in the aged brain.

## Material and Methods

### Animals

Young (3 month-old) and aged (22 month-old) adult male C57Bl/6 mice (National Institute of Aging colony, Charles River Laboratories) were housed in the Animal Care facility at the University of Maryland School of Medicine under a 12 hour light-dark cycle, with *ad libitum* access to food and water. All surgical procedures were carried out in accordance with protocols approved by the Institutional Animal Care and Use Committee (IACUC) at the University of Maryland School of Medicine.

### Controlled Cortical Impact (CCI)

Our custom-designed CCI device consists of a microprocessor-controlled pneumatic impactor with a 3.5 mm diameter tip as described (41). Briefly, mice were anesthetized with isoflurane evaporated in a gas mixture containing 70% N_2_O and 30% O_2_ administered through a nose mask. Mice were placed on a heated pad and core body temperature was maintained at 37°C. The head was mounted in a stereotaxic frame, a 10-mm midline incision was made over the skull and the skin and fascia were reflected. A 5-mm craniotomy was made on the central aspect of the left parietal bone. The impounder tip of the injury device was then extended to its full stroke distance (44 mm), positioned to the surface of the exposed dura, and reset to impact the cortical surface. Mild-level CCI was induced using an impactor velocity of 6 m/s, deformation depth of 1 mm, and a dwell time of 50 ms (8, 42). After injury, the incision was closed with interrupted 6-0 silk sutures, anesthesia was terminated, and the animal was placed into a heated cage to maintain normal core temperature for 45 minutes post-injury. Sham animals underwent the same procedure as CCI mice except for the impact. Mice were anesthetized (100 mg/kg sodium pentobarbital, I.P.) 24 hours post-injury. Mice were transcardially perfused with ice-cold 0.9% saline (100 ml). Ipsilateral cortical and hippocampal tissue were rapidly dissected and snap-frozen on liquid nitrogen for RNA or protein extraction.

### Western Blot Analysis

Proteins from ipsilateral cortical tissue of sham and TBI young and aged mice (n=4/group) were extracted using RIPA buffer, equalized, and loaded onto 5–20% gradient gels for SDS PAGE (Bio-Rad; Hercules, CA). Proteins were transferred onto nitrocellulose membranes, and then blocked for 1 hour in 5% milk in 1 × TBS containing 0.05% Tween-20 (TBS-T) at room temperature. The membrane was incubated in anti-α-Fodrin (1:1000, BML-FG6090; Enzo Life Science, Farmingdale, NY), anti-PARP (1:1000, 94885; Cell signaling, Danvers, MA), anti-phospho-c-Jun (S73) (1:1000, 3270; Cell signaling), anti-PSD95 (1:1000, D27E11; Cell signaling), anti-cGAS (1:1000, 31659; Cell signaling), anti-STING (1:500, 13647; Cell signaling), anti-pSTAT1 (1:1000, 7649; Cell signaling), anti-STAT1 (1:1000, 9172; Cell signaling), anti-pSTAT3 (1:1000, 9145; Cell signaling), anti-STAT3 (1:1000, 9139; Cell signaling), anti-p21 (1:2000, ab188224; Abcam, Cambridge, MA) or anti-β-Actin (1:5000, A1978; Sigma-Aldrich, St Louis, MO) overnight at 4°C, then washed three times in TBS-T, and incubated in appropriate HRP-conjugated secondary antibodies (Jackson ImmunoResearch Laboratories, West Grove, PA) for 2 hours at room temperature. Membranes were washed three times in TBS-T, and proteins were visualized using SuperSignal West Dura Extended Duration Substrate (Thermo Scientific, Rockford, IL). Chemiluminescence was captured ChemiDoc™ XRS+ System (Bio-Rad Laboratories, Hercules, CA), and protein bands were quantified by densitometric analysis using BioRad Molecular Imaging Software. The data presented reflects the intensity of target protein band normalized to the intensity of the endogenous control for each sample (expressed in arbitrary units).

### NanoString Analysis

Total RNA was extracted from snap-frozen sham and TBI cortical tissue from young and aged mice (n=6/group) using an RNeasy isolation kit (Qiagen, Valencia, CA) with on-column DNase treatment (Qiagen). An nCounter Mouse Immunology Panel (NanoString Technologies, Seattle, WA) was used to determine the expression levels of genes associated with innate and adaptive immunity. Each target gene of interest was detected using a pair of reporter and capture probes that together target a continuous 100 nucleotide sequence. Hybridization between 100 ng of target mRNA and reporter-capture probe pairs was performed at 65°C for 20 hours using a Perkin Elmer Thermal Cycler (Perkin Elmer, MA) according to the manufacturer’s protocol. Post hybridization processing was carried out on a fully automated nCounter Prep station liquid-handling robot. Excess probes were removed, and stable probe/target complexes were aligned and immobilized in the nCounter cartridge, which was then placed in a digital analyzer for image acquisition and data processing (nCounter Digital Analyzer). The expression level of gene transcripts was determined by directly counting the number of times the specific barcode for each gene was detected, and the barcode counts were then tabulated in a comma-separated value (CSV) format. The raw digital transcript counts (RCC files) and corresponding reporter library files (RLF) were imported into NanoString nSolver v 4.0 software for analysis of quality control parameters, determination of the raw counts for each transcript. and data normalization using standard default software settings. Raw counts were multiplied by scaling factors proportional to the sum of counts for spiked-in positive control probes to account for individual assay efficiency variation, and to the geometric average of the normally distributed housekeeping gene probes that are built into every NanoString panel. Background signal was calculated as a median value of the negative hybridization control probes. Normalized counts were log-transformed for downstream statistical analysis. One sample in the Young TBI group did not pass quality control metrics and was removed from further inclusion in the NanoString analysis. Normalized NanoString data and raw data files are publicly available on GE0 (GSE180811).

### Quantitative Real-Time PCR (qRT-PCR) Analysis

Total RNA was extracted from snap-frozen sham and TBI ipsilateral cortical and hippocampal tissue from young and aged mice (n=7-9/group) as described before. cDNA synthesis was performed using a Verso cDNA RT kit (Thermo Scientific, Pittsburg, PA) according to the manufacturer’s instructions. qRT-PCR was performed using TaqMan gene expression assays (Ifnb1, Mm00439552_s1; Irf7, Mm00516793_g1; Isg15, Mm01705338_s1; Ifi204, Mm00492602_m1; Irf1, Mm01288580_m1; Irf3, Mm00516784_m1; Irf4, Mm00516431_m1; Irf5, Mm00496477_m1; Irf7, Mm00516793_g1; Mx1, Mm00487796_m1; and Gapdh, Mm99999915_g1) on an ABI 7900 HT FAST Real Time PCR machine (Applied Biosystems, Carlsbad, CA). Samples were assayed in duplicate in one run (40 cycles), composed of 3 stages: 50°C for 2 minutes, 95°C for 10 seconds for each cycle (denaturation), and finally the transcription step at 60°C for 1 minute. Gene expression was calculated relative to the endogenous control sample (Gapdh) to determine relative expression values, using the 2−ΔΔCt method (where Ct is the threshold cycle) (43).

### Statistical Analysis

Mice that were ear tagged and housed five per cage were randomly removed one at a time from the cage and assigned to groups until sufficient numbers were reached for each group. Quantitative data were expressed as mean ± standard errors of the mean (s.e.m.). Normality testing was performed and data passed normality (D’Agostino & Pearson omnibus normality test), and therefore parametric statistical analysis was performed. Statistical analysis for Western blot and qRT-PCR experiments was carried out using a two-way analysis of variance (ANOVA) with Tukey *post-hoc* tests and were performed using GraphPad Prism Program, Version 9 for Mac (GraphPad Software, San Diego, CA, USA). Significance level was set as p<0.05. For highly variable Western blot studies we excluded samples that were more than two standard deviations from the mean of the group (n=1 only). For NanoString data, log-transformed and background normalized expression values were used in all statistical analyses. Normality of expression levels within each injury status group was assessed using the Shapiro-Wilk test to determine whether parametric tests were appropriate. Linear regression models were used to assess the relationship between expression levels and the presence of TBI while accounting for the two age groups of mice (young *versus* aged). Each model included the expression level as the dependent variable and independent variables of injury status and age group. No interaction terms were investigated. Resulting p-values for both the injury and age group were adjusted for the number of expression values compared using the Benjamini-Hochberg method to control the false discovery rate. An adjusted p-value ≤ 0.05 was considered evidence of an effect. All analyses were performed using STATA V15 (College Station, TX). For differential expression analysis with fold change, the NanoStringDiff (44) tool was used. It uses a generalized linear model of the negative binomial type to characterize the raw count data. Normalization is achieved by calculating size factor from positive control and housekeeping genes, and background levels evaluated from negative controls. Genes with log fold change base 2(log2) greater than +/- 1.5, and adjusted p-value less than 0.05, were considered significant. Pathway analysis was performed with Ingenuity Pathways Analysis (IPA) (Qiagen, Gaithersburg, MD). Gene ontology analysis was performed with gProfiler (45). Gene association networks were analyzed using GeneMANIA (46), and visualization of the networks obtained using Cytoscape (47). Visualization of heatmaps and gene ontology plots (bubble and GOChord) were generated in R. Heatmaps were built using the pheatmap (Raivo Kolde (2019). pheatmap: Pretty Heatmaps. R package version 1.0.12. https://CRAN.R-project.org/package=pheatmap) package, while gene ontology plots were generated using GO Plot (48) package.

## Results

### TBI Induces DNA Damage and Cell Death in the Cortex of Young and Aged Mice

**Figure 1:**
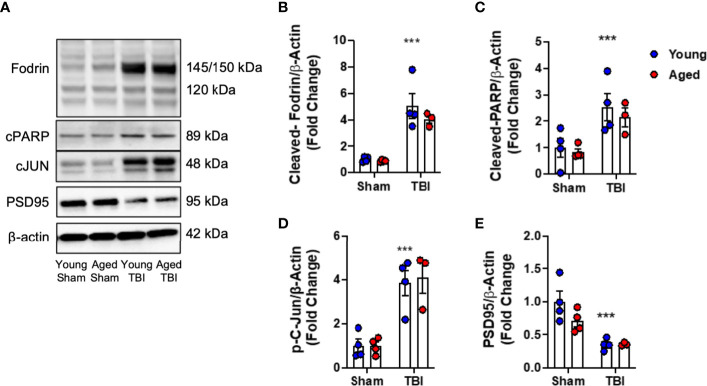
DNA damage, cell death and neuroplasticity markers in the injured cortex of young and aged mice. Western blot expression of cleaved-Fodrin **(B)**, cleaved-PARP **(C)**, phospho-c-Jun **(D)** and PSD95 **(E)** protein in the ipsilateral cortex young and aged Sham and TBI mice at 24 hours post-injury. Representative Western blots are shown in **(A)** TBI significantly increased cortical expression of cleaved-Fodrin (p<0.0001, **B**), cleaved-PARP (p=0.0075, **C**) and phosphor-c-Jun (p<0.0001, **D**) protein in young and aged mice. There was no effect of age on protein expression. TBI significantly decreased protein expression PSD95 (p<0.0001, **E**). There was no effect of age on PSD95 protein expression. Data expressed as Mean ± SEM. ***p < 0.001 *vs.* sham (effect of TBI). Two-way ANOVA using Tukey post-hoc tests (n=3-4/group).

First, we examined the effect of age on neuronal cell death pathways in injured cortical tissue. We induced mild-level CCI in young (3 month-old) and aged (22 month-old) male C57Bl/6 mice and performed biochemical analysis of markers of DNA-damage associated cell death pathways (49) at 24 hours post-injury. When compared to age-matched sham mice, TBI increased cortical levels of cleaved-Fodrin (145-150 kDa) (F_(1,11)_=327.2, p<0.0001; **Figures 1A, B** **)**, cleaved-PARP (F_(1,11)_=14.88, p=0.0027; **Figures 1A, C** **)** and phospho-c-Jun (F_(1,11)_=41.71, p<0.0001; **Figures 1A, D** **)** in young and aged mice. There was no effect of age on these pathways. In addition, we examined the expression of synaptic protein PSD95. TBI significantly decreased the protein expression of PSD95 in young and aged mice (F_(1,11)_=19.55, p<0.001; **Figures 1A, E** **)**; however, there was no effect of aged on this TBI-induced change. Thus, TBI induced significant DNA damage and neuronal cell death, and reduced postsynaptic density in young and aged mice.

### TBI Is the Main Driver of Differentially Expressed Inflammatory Genes in Young and Aged Mice

**Figure 2:**
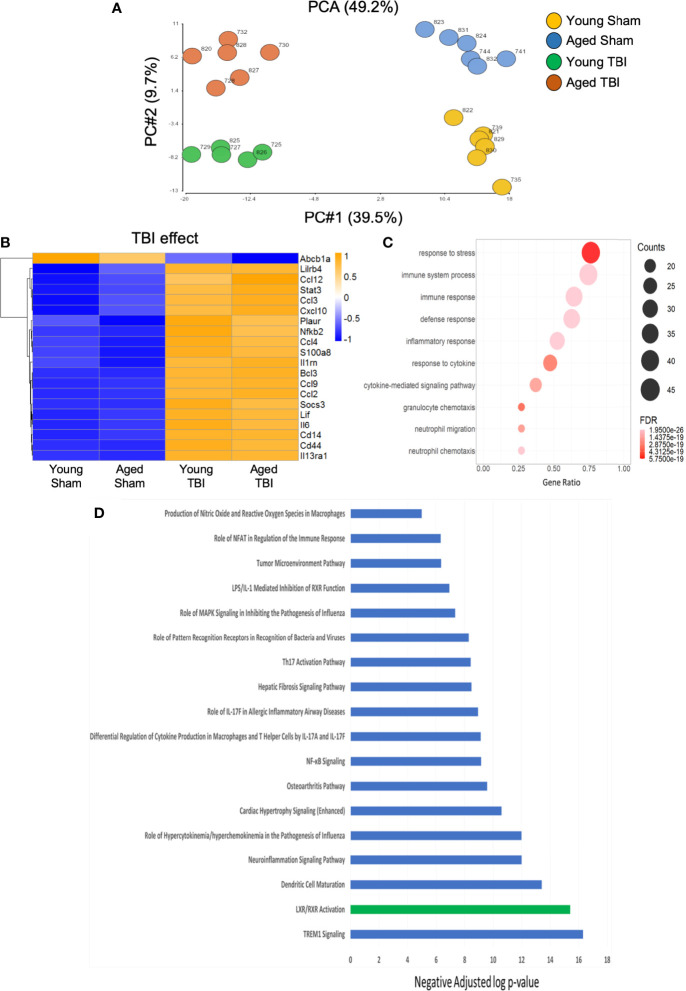
Inflammatory gene expression in injured cortex is primarily altered by TBI. NanoString analysis using a mouse Immunology panel was used to assess cortical transcriptional patterns at 24 hours post-injury in young sham, aged sham, young TBI, and aged TBI mice (n=6/group). **(A)** A principle components analysis of all transcripts by subject identifies key genes changed by TBI (PC1) and age (PC2). There was no overlap and minimal within group variability in this analysis. **(B)** Expression map of the top 20 transcripts that were significantly altered by TBI only. The heat map color scale reflects normalized log-transformed raw counts scaled to z-scores, where -1=darker blue=decreased expression and 1=darker yellow=increased expression. All transcripts that were significantly altered by TBI only are shown in **Supplemental Table 1**. **(C)** Dot plot of biological function GO terms enriched in the group of transcripts that was significantly altered by TBI only (R2>0.85), with no significant covariate effect of AGE. The color of the dots is based on adjusted p-value (FDR) with the darker color (red) corresponding to higher/greater p-value, and lighter color (violet) corresponding to lower/smaller p values. The radius of the dots is based on the number of genes assigned to the term. The x axis corresponds to the gene ratio, which is the ratio of genes within the dataset represented within a given gene ontology (GO) term and total number of genes assigned the term. There was no secondary thresholding for injury coefficients. **(D)** All transcripts that were significantly altered by injury (and the respective p-values for injury effect) were analyzed with Ingenuity Pathways Analysis to determine which canonical pathways were represented, and assign a z-score to indicate pathway activation (positive z-score) or deactivation (negative z-score). The top canonical pathways (-log p value of ≥ ≤ 5.0. with z scores >2 or <-2) are represented. All were positive (activated=blue bars), except LXR/RXR which was negatively regulated (deactivated=green bar). Individual z-scores and the genes that contribute to each canonical pathway are shown in **Supplemental Table 5**.

We then used a murine immunology NanoString panel to characterize global changes in gene expression associated with immune and inflammatory pathways in the perilesional cortex of young and aged sham and TBI mice. A principle components analysis of all transcripts by subject showed definitive separation of each study group (no overlap) and minimal within group variability (**Figure 2A**). Of 547 genes on the panel, linear regression indicated that expression levels of 236 were significantly changed, with 210 altered by TBI alone, 12 by age alone, and 14 affected by both TBI and age (**Supplemental Tables 1**–**3**). With differential expression analysis based on fold change, 193 genes were altered by TBI alone (**Supplemental Table 4**). There was extensive overlap of the differentially expressed genes represented in both young and aged TBI, compared to their respective shams. Only 14 differentially expressed genes were significantly different between young and aged TBI (**Supplemental Figure 1**).

We next performed functional analysis of discrete gene sets using gene ontology (GO) analysis. TBI-only induced changes were highly enriched for transcripts with functional GO terms involved in inflammatory responses to stress and host defense (**Figures 2B, C** **)**. Cytokine-driven responses and transcripts involved in neutrophil migration and chemotaxis were also well represented. Specific canonical pathways significantly altered by TBI alone included a strong activation of IL-17 pathways (**Figure 2D** and **Supplemental Table 5**). IL-17 is a pro-inflammatory cytokine associated with activation of IL-23/IL-17 signaling axis linked to apoptotic cell death and functional deficits after experimental TBI (50). Upregulation of lipopolysaccharide (LPS)/IL-1, an inflammatory pathway that can inhibit retinoid X receptor (RXR) activation was observed concurrent with downregulation of ligand-activated nuclear liver X (LXR) and RXR associated transcripts. These complementary data provide strong support for an inflammation-associated down regulation of LXR/RXR signaling after TBI. Agonists of LXR/RXR have anti-inflammatory properties and improve Aβ clearance and reduce plaque development in mouse models of AD, where they also reduced memory deficits and preserved expression of synaptic components (51). The down-regulation of LXR/RXR observed here could contribute to pathological aspects of TBI that replicate the pathophysiology of AD. In fact, we have previously demonstrated that LXR agonists have neuroprotective properties and improve functional recovery after TBI in mice (52). Activation of signaling associated with viral or bacterial infection and host response (NFkB, MAPK, PRRs) was also evident and consistent with enriched functions from the GO term analysis.

**Figure 3:**
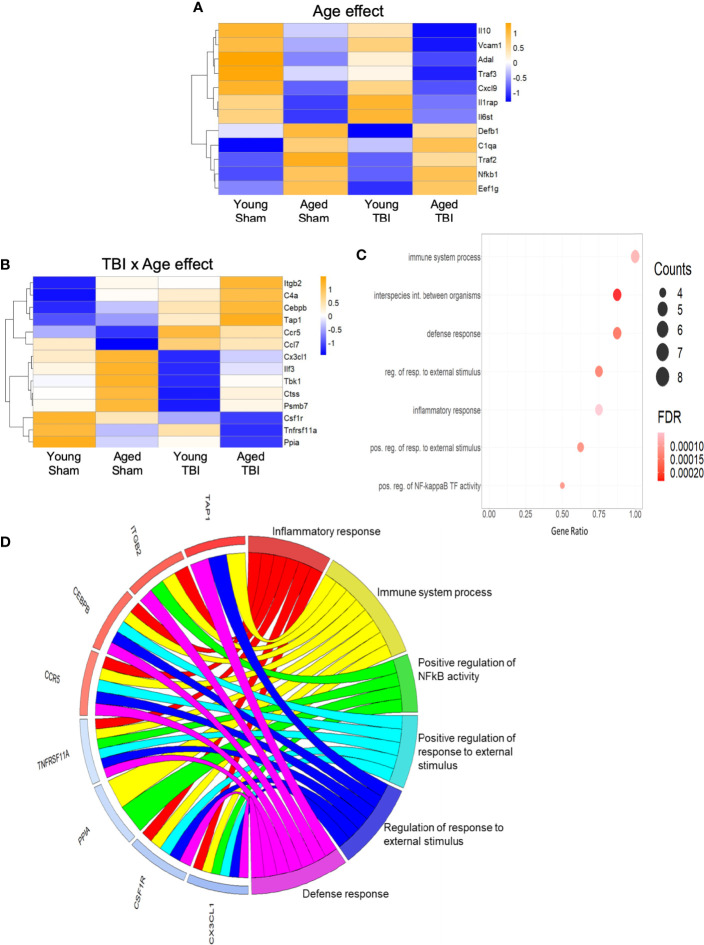
Inflammatory gene expression in injured cortex is also altered by age alone or combined aged plus TBI. **(A)** Expression map of transcripts that were significantly altered by age only. Of these 12, only 4 had R2 values of >0.80 (**Supplemental Table 2**), indicating that the expression of this group contributed only minimally to the regression model. Biological function GO terms could not be assigned to this group, due to their small number. The heat map color scale reflects normalized log-transformed raw counts scaled to z-scores, where -1=darker blue=decreased expression and 1=darker yellow=increased expression. **(B)** Expression map of transcripts that were significantly altered by TBI and age (14 genes, 9 with R2 >0.80). The heat map color-scale reflects normalized log-transformed raw counts scaled to z-scores, where -1=darker blue=decreased expression and 1=darker yellow=increased expression. **(C)** Dot plot of biological function GO terms enriched in the group of transcripts that was significantly altered by TBI and age (R2>0.85). The list of individual genes and their significance values appears in **Supplemental Table 3**. The color of the dots is based on adjusted p-value (FDR) with the darker color (red) corresponding to higher/greater p-value, and lighter color (violet) corresponding to lower/smaller p values. The radius of the dots is based on the number of genes assigned to the term. The x axis corresponds to the gene ratio, which is the ratio of genes within the dataset represented within a given GO term and total number of genes assigned the term. There was no secondary thresholding for injury coefficients. The term “interspecies int. between organisms” is abbreviated from the full formal name = biological process involved in interspecies interaction between organisms, “reg. of resp. to external stimulus” abbreviated from = regulation of response to external stimulus, “pos. reg. of resp. to external stimulus” for = positive regulation of response to external stimulus, and “pos. reg. of NF-kappa B TF activity” for = positive regulation of NF-kappaB TF activity. **(D)** CIRCOs plot shows the involvement of genes from **(B)** with multiple roles in the biological functions defined in **(C)**. The red and blue bars next to each individual gene indicate increased (red) or decreased (blue) expression of the gene, where intensity of shading indicates the relative change (dark/greater to light/less). Cebpb, Cx3cl1, Ccr5 and Csf1r have a wide range of functions, and were associated with 5 of the 6 GO terms.

Only 12 transcripts were altered by age alone and no clear consistent pathway or GO terms could be strongly associated with these (**Figure 3A**), likely in part due to the limited number of genes represented. Fourteen transcripts were altered by both TBI and age (**Figure 3B**), and the effect of age was variable, with some transcripts increased and others decreased. Several of these are known to play important roles as regulators of inflammation and immune defense responses (**Figure 3C**). For example, CCAAT/Enhancer binding protein beta (Cebpb) is a transcription factor that regulates downstream expression of multiple pro-inflammatory genes. Cebpb was increased in aged sham compared to young sham mice and still further increased by TBI, with highest levels after TBI in aged mice. These data agree with those indicating an in increase in Cebpb protein in aged animals after experimental TBI (53). The expression of colony stimulating factor 1 receptor (Csf1r) was decreased in aged sham compared to young sham mice, and was further decreased in aged TBI mice compared to young TBI mice. Csf1r is essential for microglial survival, and Csf1r inhibitors that promote microglial depletion during the chronic phase after TBI improve outcomes (54). The protective effect of Csf1r inhibitors following TBI is likely due to the removal of neurotoxic microglia, because microglia that repopulate the CNS exhibit diminished pro-inflammatory activity (54). Recent single cell transcriptomics studies revealed that expression of Csf1r in microglia clusters with other homeostatic genes such as P2ry12 and Cx3cr1 (55). Decreased expression of Csf1r with age and TBI may reflect a reduction in the proportion of homeostatic microglia.

The C-X3-C motif chemokine ligand 1 (Cx3cl1; fractalkine) is primarily expressed by neurons, where it interacts with the Cx3cl1 receptor on microglia to maintain them in a quiescent/inactivated state (56). It is possible that the increase in Cx3cl1 observed with aging may reflect chronic homeostatic upregulation to protect against activation of microglia during normal aging. The decrease in Cx3cl1 after TBI may reflect injury-induced loss of the neurons that produce Cx3cl1, or a decrease in Cx3cl1 synthesis by intact neurons that would result in enhanced activation of microglia following TBI (56). The potential of these important inflammatory regulators (Cepbp, Csf1r, Cx3cl1) as well as several others (Itgb2, Ccr5, Tnfrsf11a) that have multipotent roles in inflammatory processes is depicted in a CIRCOs plot (**Figure 3D**). This analysis shows the association of these individual transcripts with GO terms for a broad range of inflammatory/immune defense functions.

**Figure 4:**
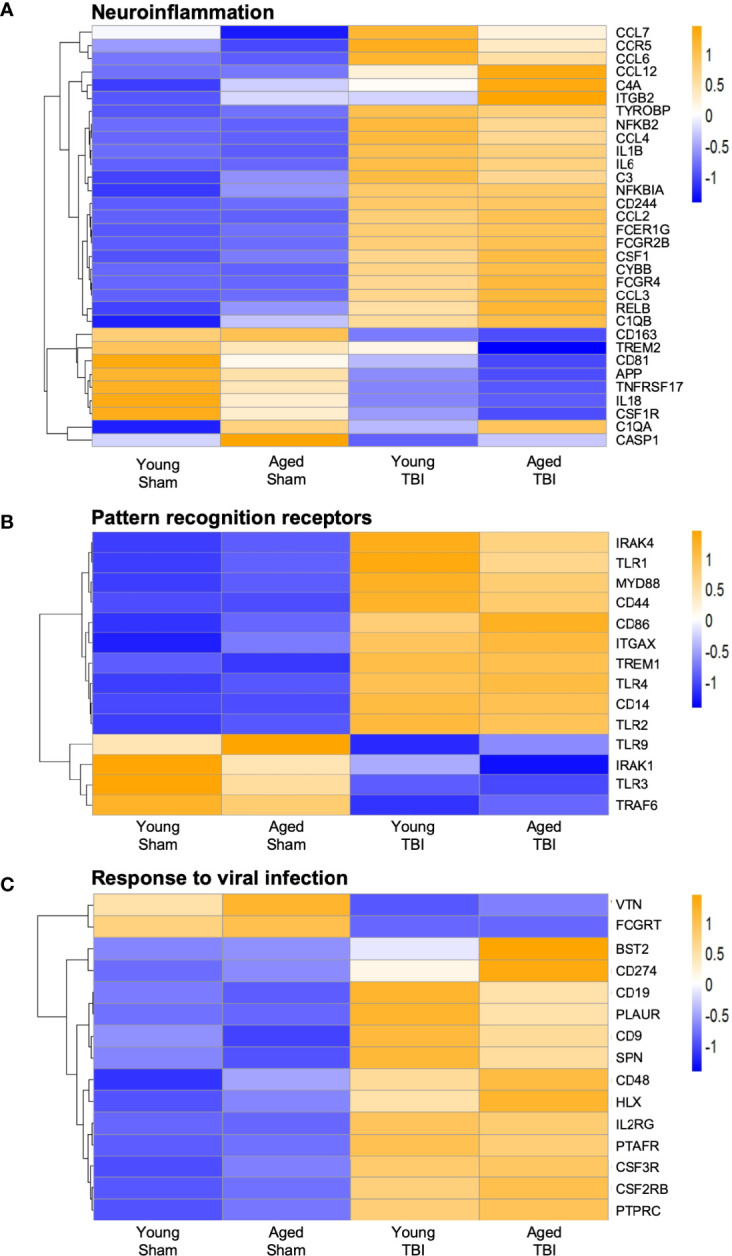
Expression of selected genes related to neuroinflammation, pattern recognition receptor, and response to viral infection. Selected transcripts were grouped by known or suspected functions related to neuroinflammation**(A)**, pattern recognition receptors **(B)** and viral infection **(C)**. Blue (cool colors) were used to graph low expression levels and orange (warmer colors) depict higher expression levels. For A-C, the heat map color scale reflects normalized log-transformed raw counts scaled to z-scores, where -1=darker blue=decreased expression and 1=darker yellow=increased expression. For most but not all transcripts, TBI increases expression. For some transcripts, TBI in aged mice shows the highest level of expression, but this age effect was not statistically significant for any transcript in the TBI only group.

We next focused on genes that are classically linked to neuroinflammation **(** **Figure 4A** **)**. Significantly, a number of genes associated with pro-inflammatory responses, including cytokines (Il1b, Il6), chemokines (Ccl2, Ccl3), complement pathway (C1qa, C1qb, C3, C4a), NFkB signaling (Relb, Nfkbia, Nfkb2) and NOX2 activity (Cybb) were upregulated with TBI and aging. Of note, NOX2 inhibition improves neurological recovery after TBI and reduces chronic neurodegeneration (57, 58). The age-related increase in the expression of genes associated with the complement system agrees with prior reports suggesting this pathway is enhanced in aging (59–61). There was also increased expression of a number of genes linked to DAMs (Csf1, Tyrobp, Ccl6) (55). For down-regulated genes, Trem2 expression was decreased by age and TBI. The genetic associations of Trem2 loss-of-function variants (e.g. R47H) with AD and other forms of dementia highlight the essential role of microglial Trem2 in maintaining homeostasis in brain (62).

When we reviewed PRRs we found a significant increase in TLR gene expression, such as Tlr1, Tlr2 and Tlr4, and increased expression of downstream signaling genes such as Irak4 and Myd88 **(** **Figure 4B** **)**. Although classically associated with the recognition of invading pathogens, preclinical studies demonstrate that inhibition of TLR2 and TLR4 is neuroprotective following TBI (63, 64). There was also a significant decrease in the expression of Tlr9 and Tlr3, suggesting differential regulation of TLRs following TBI. Overall, the data demonstrate that TBI results in upregulation of genes associated with neuroinflammation and microglial reactivity to DAMPs.

### Viral Infection and Type I Interferon Pathways Are Upregulated in Aged TBI Mice

NanoString analysis also identified genes associated with increased viral infection in young and aged TBI mice when compared to age-matched sham mice **(** **Figure 4C** **)**. These viral genes included Bst2, CD274 and Hlx. Notably, various genes traditionally associated with viral responses have been reported to be upregulated after CNS injury and during chronic neurodegeneration (2, 6, 17, 65). Given this viral response signature and the recent identification of IFN-I signaling in TBI pathobiology, we next investigated if there were any age-related changes in IFN-I genes following TBI.

**Figure 5:**
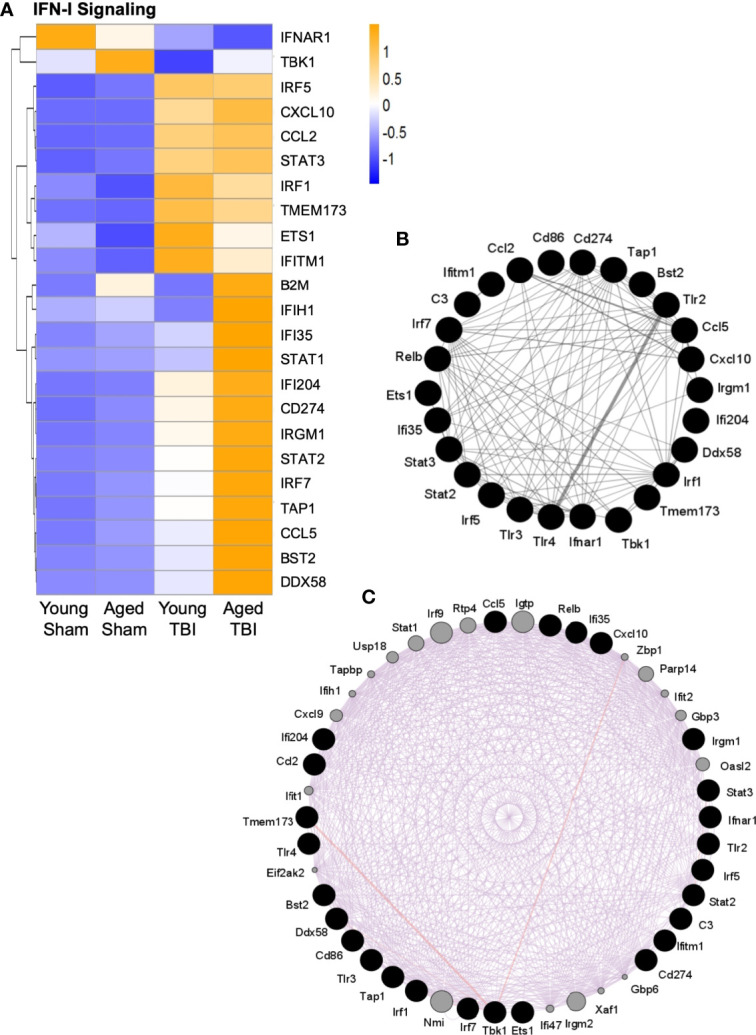
Interaction analysis of selected genes related to IFN-I signaling. Selected transcripts were grouped by known or suspected functions related to IFN-I signaling **(A)**. For most but not all transcripts, TBI increases expression. For some transcripts, TBI in aged mice shows the highest level of expression, and this age effect was statistically significant for several transcripts. The heat map color scale reflects normalized log-transformed raw counts scaled to z-scores, where -1=darker blue=decreased expression and 1=darker yellow=increased expression. **(B, C)** GeneMania (http://www.genemania.org) was used to generate an expression interaction between genes represented in **(A)**. Black nodes are the query genes (from A) and the connections/edges are colored based on interaction type, where **(B)** grey is for interactions between genes whose expression was altered **(A, C)** purple is for predicted interactions between genes in **(A)** (black nodes) and other genes (grey nodes) based on literature citations. The width of the edges is controlled by the evidence of the interaction, where thicker edges denote more evidence of interaction between the attached genes/nodes. Specific interaction values for each gene are shown in **Supplemental Table 6**.

A number of genes classically associated with the IFN-I response were also elevated in the young and aged TBI brain **(** **Figure 5A** **).** Stat1, Stat2 and Stat3 genes, three downstream transcription factors associated with IFN-I signaling (66), were increased following TBI, and both Stat1 and Stat2 gene expression were further increased in aged TBI mice. Irf1, Irf5 and Irf7, three IRF family members that are implicated in pro-inflammatory responses in macrophages (32), were increased in young and aged TBI mice. Consistent with previous reports that Irf7 is increased with advanced age (13), we observed that Irf7 expression was highest in the aged TBI group. In addition, the expression of a number of genes associated with IFN-I responses were also increased in aged TBI mice, including Ifi35, Irgm1, Ifi204, Ifih1, and Ddx58. Significantly, Ifi204, Ifih1 and Ddx58 are also involved in the detection of nucleic acids and lead to induction of IFN-I (67–70). Interestingly, Tbk1, Tlr3 and Ifnar1, all of which contribute to IFN-I signaling, were downregulated following TBI, which could be a compensatory mechanism following TBI.

As multiple transcripts related to IFN-I signaling were activated by TBI (**Figure 5A**), it was possible to evaluate these for potential interactions. The GeneMANIA tool was used to search publicly available primary data from biological datasets (BioGrid, PathwayCommons) that identify physical and/or functional interactions between genes. Multiple specific interactions were supported among the IFN-I signaling transcripts that are altered by TBI (**Figure 5B**). Additional potential interactors were also predicted (**Figure 5C** and **Supplemental Table 6**). These included IFN-I signaling genes that were not on the NanoString immunology panel used here, such as z-DNA-binding protein 1 (Zbp1), poly(ADP-ribose)polymerase 14 (PARP14), Ubiquitin-specific peptidase 18 (USP18), IRF9 and interferon-induced, double-stranded RNA-activated protein kinase (eif2AK2, also known as PKR). To our knowledge, these have not previously been associated with TBI, although they are established participants in IFN-I antiviral responses. Zbp1 is a nucleic acid sensing pattern recognition receptor that promotes inflammation and cell death *via* the RIPK1/RIPK3–FADD–caspase-8 pathway (71). IRF9 shares homology with other IRF proteins and similarly regulates IFN-stimulated gene expression, in part through STAT proteins (72). PKR promotes inflammation by activating NLRP1, NLRP3 and AIM2 inflammasomes (73, 74). In contrast, USP18 is a negative regulator of IFN-I signaling (75) and PARP14 may have anti-inflammatory properties by limiting STAT activation (76).

### TBI Induces an Enhanced IFN-I Gene Signature in the Cortex and Hippocampus of Aged Mice

**Figure 6:**
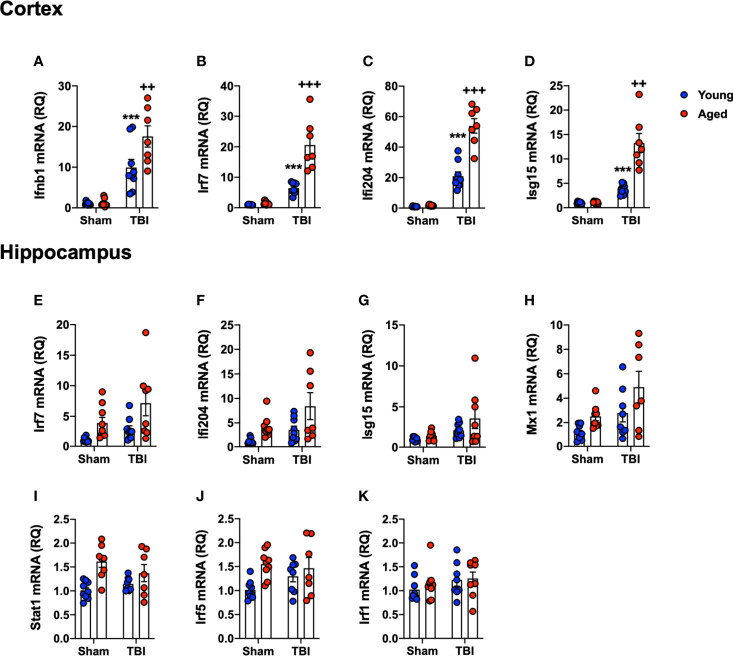
Confirmation analysis of IFN-I genes the cortex and hippocampus of young and aged sham and TBI mice. Cortical and hippocampal expression of genes associated with IFN-I signature were assessed in young and aged sham and TBI at 24 hours post-injury. In the cortex, TBI significantly increased mRNA expression of Ifnb1 (p<0.0001, **A**), Irf7 (p=0.0014, **B**), Ifi204 (p<0.0001, **C**) and Isg15 (p=0.0002, **D**) in young and aged mice. The TBI-induced increase in Ifnb1, Ifi204, Irf7 and Isg15 was significantly elevated in the cortex of aged mice. In the hippocampus, TBI significantly increased expression of a number of IFN-I related genes in young and aged mice, Irf7 (p=0.0105, **E**), Ifi204 (p=0.0133, **F**), Isg15 (p=0.0135, **G**) and Mx1 (p=0.0078, **H**). Age also significantly increased expression of Irf7 (p=0.005, **E**), Ifi204 (p=0.0028, **F**), and Mx1 (p=0.0041, **H**), but there was no significant interaction between age and TBI. Age significantly increased the expression of Stat1 (p=0.0006, **I**) and Irf5 (p=0.0091, **J**), but there was no effect of TBI on either gene. No effect of age or TBI was observed on Irf1 mRNA expression **(K)**. Data expressed as Mean ± SEM. ***p < 0.001 *vs.* sham (effect of TBI) and ^++^p < 0.01, ^+++^p < 0.001 young TBI *vs* aged TBI. Two-way ANOVA using Tukey post-hoc tests, (n=7-9/group).

As a complimentary method to support the NanoString data, and to focus on our interest in IFN-I responses, we assessed the expression of selected genes in the IFN-I pathway by qRT-PCR. RNA from ipsilateral cortex and hippocampus of sham and TBI young (3 month-old) and aged (22 month-old) mice was used in this analysis. We first quantified IFN-β because we had previously identified it to be an important driver of secondary neuroinflammation after TBI (11). TBI significantly increased Ifnb1 mRNA expression in young and aged mice (F_(1,30)_=69.72, p<0.0001; **Figure 6A**). There was a significant age effect (F_(1,30)_=6.571, p=0.0156), and there was also a significant interaction between TBI and age (F_(1,30)_=6.118, p=0.0193). Post-hoc analysis revealed an age-related increase in cortical Ifnb1 mRNA expression in aged TBI mice (p=0.0034, young TBI *vs.* aged TBI). We next evaluated the expression of a number of IFN-dependent genes in the cortex, including Irf7 (**Figure 6B**), Ifi204 (**Figure 6C**) and Isg15 (**Figure 6D**) mRNAs. TBI robustly increased the mRNA expression of Irf7 (F_(1,30)_=84.98, p<0.0001), Ifi204 (F_(1,30)_=215.4, p<0.0001) and Isg15 (F_(1,30)_=17.76, p=0.0002). There was a significant age effect on Irf7 (F_(1,30)_=29.85, p<0.0001), Ifi204 (F_(1,30)_=47.09, p<0.0001), and Isg15 (F_(1,30)_=9.96, p=0.0036), and a significant interaction of TBI and age for all genes (Irf7 (F_(1,30)_=26.43, p<0.0001); Ifi204 (F_(1,30)_=42.95, p<0.0001); Isg15 (F_(1,19)_=9.699, p=0.004)). Post-hoc analysis revealed a significant increase in Irf7, Ifi204 and Isg15 mRNA expression in aged TBI mice (Irf7, p<0.0001; Ifi204, p<0.0001; Isg15, p=0.0003; young TBI *vs.* aged TBI).

We had previously shown IFN-I pathway activation at more distant sites from the primary lesion, such as the ipsilateral hippocampus (11). Therefore, we measured IFN-related gene expression levels in the hippocampus [Irf7 (**Figure 6E**), Ifi204 (**Figure 6F**), Isg15 (**Figure 6G**), Mx1 (**Figure 6H**), Stat1 (**Figure 6I**), Irf5 (**Figure 6J**) and Irf1 (**Figure 6K**)]. TBI increased hippocampal expression of IFN genes at 24 hours post-injury (Irf7, F_(1,30)_=5.167, p=0.0105; Ifi204, F_(1,30)_=6.917, p=0.0133; Isg15, F_(1,30)_ =6.899, p=0.0135: Mx1, F_(1,30)_=8.205, p=0.0078). There was a significant age effect on Irf7 (F_(1,30)_=13.87, p=0.0008), Ifi204 (F_(1,30)_=9.824, p=0.0038) and Mx1 (F_(1,30)_=6.207, p=0.0189); however, there was no interaction between TBI and age. While there was no effect of TBI on Irf5 or Stat1, age alone significantly increased the expression of these genes (Irf5, F_(1,30)_=7.810, p=0.0091; Stat1, F_(1,30)_=14.72, p=0.0006). There was no effect of age or TBI on Irf1 mRNA expression (**Figure 6K**). Overall, these data confirm the NanoString analysis, and demonstrates that TBI in aged animals increases IFN-I signature genes in both the cortex and hippocampus.

### TBI Induces Age-Related Activation of cGAS Pathway in the Injured Cortex

**Figure 7:**
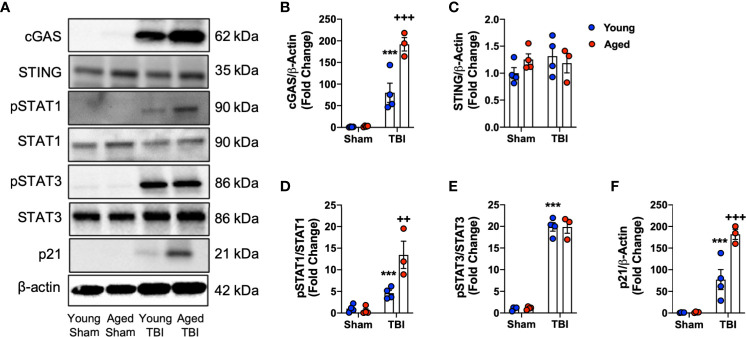
Biochemical analysis of cGAS/STING and IFN-I signaling pathways in the cortex of young and aged sham and TBI mice. Cortical protein expression of cGAS, STING and other downstream mediators of IFN-I signaling was assessed by Western immunoblotting in young and aged Sham and TBI mice at 24 hours post-injury. Representative Western blots are shown in **(A)**. TBI increased expression of cGAS (p<0.0001, **B**) protein compared to sham mice. The TBI effect was significantly increased in aged mice (cGAS p=0.0007, young TBI *vs* aged TBI). There was no effect of TBI or age on STING **(C)** protein expression. The expression of pSTAT1 protein was significantly increased in the cortex of TBI mice compared to sham mice (p=0.001, **D**). The TBI effect was significantly increased in aged mice (p=0.0146, young TBI *vs* aged TBI). The expression of pSTAT3 protein was significantly increased in the cortex of TBI mice compared to sham mice (p<0.0001, **E**). There was no age-related effect observed. TBI significantly increased p21 expression in young and aged mice (p<0.0001, **F**). The TBI effect was significantly increased in aged mice (p<0.0001, young TBI *vs* aged TBI). Data expressed as Mean ± SEM. ***p < 0.001 *vs.* sham (effect of TBI) and ^++^p < 0.01, ^+++^p < 0.001 young TBI *vs* aged TBI. Two-way ANOVA using Tukey post-hoc tests, (n=3-4/group).

Having observed enhanced expression of IFN-I related genes in the aged brain by NanoString, Western blotting was performed to analyze the activation of pathways related to IFN-I activity. Previously, we demonstrated that the cGAS/STING pathway is activated acutely after TBI (11), and activation of cGAS and STING induces the production of IFN-I (24, 77). Using ipsilateral cortical tissue from sham and CCI young (3 month-old) and aged (22 month-old) collected at 24 hours post-injury we found that TBI leads to a robust induction in cGAS protein expression (F_(1,11)_=98.57, p<0.0001; **Figures 7A, B** **)**. There was a significant age effect (F_(1,11)_=17.61, p=0.0015), and a significant interaction between TBI and age (F_(1,11)_=16.71, p=0.0018). Post-hoc analysis revealed significantly increased cGAS protein expression in aged TBI mice (p=0.0007, young TBI *vs.* aged TBI). In contrast, no effect of TBI or age was observed for STING expression (**Figures 7A, C** **)**.

STAT1 and STAT3 are two key downstream effector molecules of IFN-I signaling (78, 79). We determined that TBI increased cortical expression of pSTAT1 (**Figures 7A, D** **)** and pSTAT3 (**Figures 7A, E** **)** in young and aged mice (pSTAT1, F_(1,11)_=39.62, p<0.0001; pSTAT3, F_(1,11)_=557.9, p<0.0001). There was a significant effect of age on pSTAT1 expression (F_(1,11)_=10.24, p=0.0084) and a significant interaction between TBI and age (F_(1,11)_=12.77, p=0.0044). Post-hoc analysis revealed significantly increased pSTAT1 expression in aged TBI mice (p=0.0035, young TBI *vs.* aged TBI). There was no effect of age on pSTAT3 signaling. We also examined the expression of p21^cip1a^, which is associated with IFN-I responses and control of cell cycle (80, 81). TBI increased cortical expression of p21^cip1a^ (**Figures 7A, F** **)** in young and aged mice (F_(1,11)_=92.55, p<0.001). There was a significant age effect on p21^cip1a^ expression (F_(1,11)_=15.59, p=0.00238) and a significant interaction between TBI and age (F_(1,8)_=15.16, p=0.0025). Post-hoc analysis revealed significantly increased p21^cip1a^ expression in aged TBI mice (p=0.0146, young TBI *vs.* aged TBI). Overall, these data demonstrate that cGAS is activated in an age-dependent manner and that IFN-I associated responses (e.g. p21^cip1a^) are amplified in the aged cortex following TBI.

## Discussion

Age is a major risk factor for worsened overall outcomes following TBI (82). In the preclinical setting, we, and others, have demonstrated age-related differences in the neuroimmune response to TBI (8–10), which is characterized by enhanced inflammatory gene expression and dysregulated microglial activation. This can result in increased neurodegeneration and exaggerated neurological impairments in injured animals. However, the mechanisms that drive age-related inflammatory changes remain unknown. IFN-I contribute to secondary neuroinflammation after TBI and inhibiting this pathway provides significant neuroprotection (11, 12, 17). IFN-I have also been implicated in the development of age-related neurodegenerative diseases (14, 18, 83). Our data demonstrates that TBI in aged mice is associated with an enhanced IFN-I signature during the acute phase post-injury, which may contribute to the detrimental neuroinflammatory response and neurological deficits observed in aged TBI animals.

DNA released from dying cells can act as an alarmin, resulting in the activation of the immune response (77). Previously we, and others, demonstrated that the cGAS/STING pathway, a critical component of the cytosolic DNA sensing machinery, is robustly activated following TBI (11, 17). An interesting finding in this study is that genes associated with pathways that are involved in recognition of foreign RNA molecules are also elevated following TBI. Sensing of viral RNA is linked with the induction of IFN-I signaling (84–86). Ifih1(MDA5) and Ddx58 (RIG-I) are robustly elevated following TBI and the expression levels of both genes are significantly greater in aged TBI mice. RIG-I has been shown to contribute to the inflammatory response in the brain following cerebral ischemia and Japanese encephalitis virus infection (87–89). This raises the intriguing possibility that RNA released from dying cells may also play a role in promoting inflammatory processes in the injured brain; here, we provide evidence that this may be exaggerated in the aged TBI brain.

Our hypothesis was that increased cell death in aged TBI animals would result in excessive activation of cGAS/STING signaling. While there was no effect of age on DNA damage and activation of cell death markers, the effect of TBI on cGAS protein expression was significantly increased in the injured cortex of aged compared to young mice. A recent preclinical study demonstrated that pharmacological inhibition of cGAS following ischemic stroke in young mice reduced neuroinflammation and tissue loss (16). Interestingly, we found that STING protein expression was not altered at 24 hours post-injury, consistent with a prior study (17). However, we previously observed TBI-induced increase in STING protein expression at more delayed time points (72 hours post-injury) (11). Others have shown that Sting (Tmem173) mRNA is induced within the first 24 hours after TBI (17), which we confirm in this study. These data suggest that translation of STING protein occurs later than cGAS, or raises the possibility that the later increase in STING protein expression could be a result of peripheral immune cell infiltration following TBI. Notably, prior studies have demonstrated that peripherally-derived brain macrophages contribute to IFNAR signaling and IFN-I responses in the CNS acutely after TBI (12). While there may be no effect of age on STING expression, a previous report demonstrated that age was associated with a gain of function in STING activity (90). Therefore, while we observe no change in STING protein expression, STING activity may be enhanced in the aged TBI brain. Alternatively, the TBI-induced increase in cGAS may be independent of STING activation. In support of this, it has been shown that cGAS is essential for cellular senescence *via* a STING independent mechanism (91). We previously demonstrated that TBI in aged mice increased microglial expression of senescence markers, including p21^cip1a^ and p16^ink4a^ (8). Here, we confirmed that p21^cip1a^ expression after TBI was significantly greater in aged compared to young mice. Future studies will investigate whether age-related alterations in microglial senescence following TBI is due to increased cGAS activation.

We also determined that there was an age-related increase in phosphorylation of STAT1, a key mediator of IFN-I responses (78), following TBI. There were no age-related alterations in STAT3 activation after TBI, and previous work in immune cells has shown STAT3 limits pro-inflammatory activity of IFN-I by promoting anti-inflammatory responses (79, 92, 93). This is intriguing because it suggests that TBI in aged animals leads to an increased activation of pathways associated with pro-inflammatory IFN-I responses, while anti-inflammatory pathways, such as STAT3, are unaltered.

There is mounting evidence that IFN-I propagate inflammatory processes during normal aging (94, 95). Baruch and colleagues demonstrated that inhibition of IFN-I signaling alleviated the cognitive decline observed in aged mice (2). This is reflected in human microglia, where RNAseq analysis identified that aged microglia have a distinct phenotype, including expression of several IFN-I pathway genes such as Irf7 and Sting (Tmem173) (96). One of the most interesting findings in the current study is that while there was no evidence of enhanced activation of cell death pathways, the induction of IFN-I genes was significantly elevated following TBI. This suggests defective regulation of IFN-I in aged animals, rather than increased induction due to hyperactivated DNA damage or cell death responses after TBI. In support of this, our data show that IFN-I genes are elevated in the cortex and hippocampus of uninjured aged animals. These include a number of genes involved in the induction of IFNs, such as Tbk1. Interestingly, we found that the expression of Trem2 was decreased in aged TBI only. This may be significant because loss of Trem2 has been shown to increase IFN-I signaling (97). While IFN-I have been shown to be detrimental in CNS, it is important to reemphasize their critical role in homeostasis (98), where loss of IFN-I decreases capabilities to mount an effective immune response following infection (99). Based on our initial observations and other recent studies (11, 12, 17), investigation of TBI-induced IFN-I responses in aged animals is an important area for further investigation.

IFN-I play a significant role in age-related neurodegenerative diseases (13, 18, 28). There is increased expression of IFN-I and IFN-related genes in brain tissues across a number of mouse models of AD (18, 19, 83). Deletion of IFNAR reduces neuroinflammation and leads to improved cognitive function in the APP/PS1 mouse of AD (18). In the 5xFAD model, it has been shown that IFN-β promotes neuroinflammation and neurodegeneration (19). Notably, microglia that surrounded amyloid plaques exhibited increased expression of IFN-I genes. Our data demonstrates that IFN-related genes are increased in the uninjured aged brain, and that following TBI there is an enhanced IFN-I signature within the aged brain. Interestingly, increased activation of IFN-I signaling represents a key difference in response to ischemic stroke between young and aged mice (100), where microglia and oligodendrocytes appear to be the primary cellular source of the upregulated IFN-I signature.

Our study has several limitations that are important to discuss. One of these is that the transcriptional analysis by NanoString was carried out on cortical tissue, and thus we are unable to identify the cellular populations responsible for the IFN-I gene signatures. A recent study using a CCI model in young adult mice indicates that neurons release IFN-β in response to TBI which leads to activation of colocalized microglia (101). In contrast, Karve and colleagues provided evidence that peripheral immune cells drive the IFN-I response in the brain following TBI (12), while another recent RNAseq study identified unique microglial and astrocyte transcriptomes enriched for IFN-I following lateral fluid percussion injury in mice (102). Almost all cell types in the body can produce IFN-I (103), and it is likely that all damaged cells release IFN-I in the brain following TBI. What is clear, from our study and others, is that following TBI, IFN-I play a role in promoting pro-inflammatory neuroinflammatory responses. Future studies will focus on pinpointing the cellular source of IFN-I and identifying molecular targets that can modulate the microglial response to IFN-I stimulation after TBI.

Another limitation is that our preclinical aging study was performed in male C57Bl/6 mice so analysis of important sex differences was not possible. We previously identified sex differences in neuroimmune responses after TBI in young (3 month-old) male and female mice, whereby males had a more robust inflammatory activation acutely after TBI mediated by a rapid infiltration of pro-inflammatory macrophages to brain and robust proliferation of brain resident microglia (104, 105). The described sex differences may be age-dependent because aged females do not experience the same neuroprotection as young females (106). The underlying mechanisms are unknown, but findings from the related field of ischemic stroke indicates that this might be associated with a more pro-inflammatory microglial activation phenotype and decreased sex hormones (e.g. estrogens) in aged females (106). Further studies of age by sex interactions are clearly needed to advance basic understanding of the neuroimmunology of TBI; this is a critical next step towards developing personalized and effective treatments for TBI (107).

In summary, in this preclinical study we demonstrate that TBI in aged animals induces a robust neuroinflammatory response that is associated with increased activation of cGAS and expression of IFN-related genes. These findings identify cGAS pathway activation and IFN-I signaling as important therapeutic targets for TBI that may alleviate age-related complications following acute and chronic brain injury.

## Data Availability Statement

The raw data supporting the conclusions of this article will be made available by the authors, without undue reservation. Normalized NanoString data are publicly available on GEO (GSE180811).

## Ethics Statement

All surgical procedures were carried out in accordance with protocols approved by the Institutional Animal Care and Use Committee (IACUC) at the University of Maryland School of Medicine.

## Author Contributions

JB contributed to study design, performed *in vivo* studies, collected data, performed data analysis, and manuscript preparation. SK contributed to study conception and design, and manuscript preparation. SB performed analysis and figure preparations. HG-D performed statistical modeling and analysis. BS contributed with experimental design. DL contributed to study conception and design, manuscript preparation, and funding. All authors contributed to the article and approved the submitted version.

## Funding

This work was supported, in part, by NCMRR-DC Core for Molecular and Functional Outcome Measures in Rehabilitation Medicine NICHD R24HD050846 (DL/SK), NIH R01NS082308 (DL), NIH R01NS110756 (DL/BS), NIH R01NS096002 (BS), U.S. Veterans Affairs grant 1I01 RX001993 (BS), and Science Foundation Ireland grant 17/FRL/4860 (DL).

## Conflict of Interest

The authors declare that the research was conducted in the absence of any commercial or financial relationships that could be construed as a potential conflict of interest.

## Publisher’s Note

All claims expressed in this article are solely those of the authors and do not necessarily represent those of their affiliated organizations, or those of the publisher, the editors and the reviewers. Any product that may be evaluated in this article, or claim that may be made by its manufacturer, is not guaranteed or endorsed by the publisher.

## Supplementary Material

Supplementary Figure 1 Venn diagram of the number of genes with significantly altered expression between study groups by NanoString Diff fold-change analysis. Blue circle depicts the total number of genes that were significantly altered in the comparison of Young Sham (YSham) *vs* Young TBI (YTBI). Yellow circle shows the total number of genes significantly altered in the comparison of Aged Sham (ASham) *vs* Aged TBI (ATBI). Green circle shows genes that were significantly altered in the comparison of Young TBI *vs* Aged TBI. A total of 193 genes were altered by TBI compared to Sham (**Supplemental Table 4**), with the majority (137) altered by TBI in both young and aged animals. For other divisions within the Venn, the genes within each group are designated as follows: # = C7, Ccl20, IL23a; *= Nox4, Ccl5; ¶ = Aire, Camp, Cxcl13, Ifna1, II5, Marco, Pdcd1; & = Defb1, Fcamr.
