# Supplementary figures and images for: Traumatic Brain Injury Induces cGAS Activation and Type I Interferon Signaling in Aged Mice
Source: Front Immunol. 2021 Aug 24;12:710608. doi: 10.3389/fimmu.2021.710608 (PMC8423402; doi:10.3389/fimmu.2021.710608)

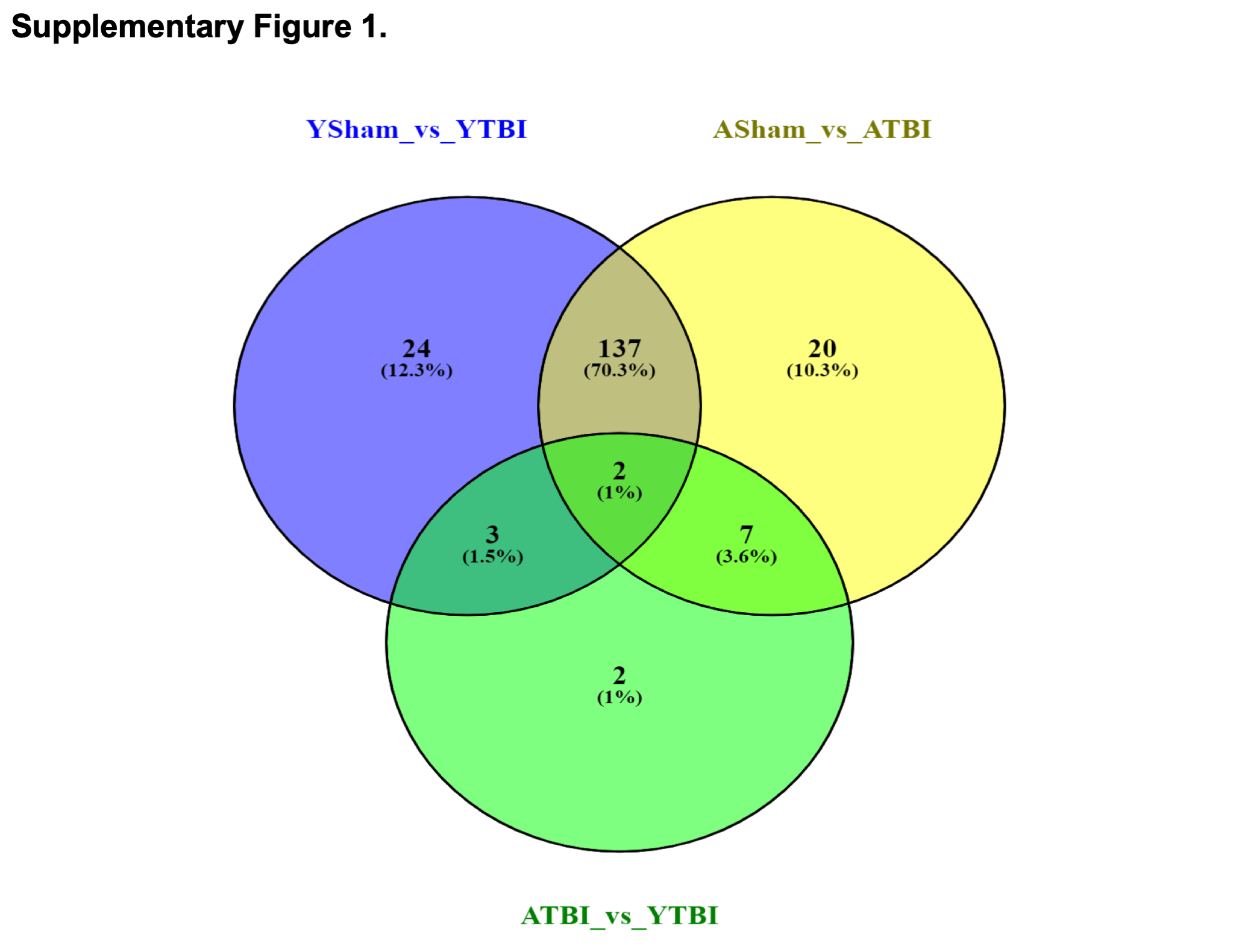

Supplement: Supplementary Figure 1 — Venn diagram of the number of genes with significantly altered expression between study groups by NanoString Diff fold-change analysis. Blue circle depicts the total number of genes that were significantly altered in the comparison of Young Sham (YSham) vs Young TBI (YTBI). Yellow circle shows the total number of genes significantly altered in the comparison of Aged Sham (ASham) vs Aged TBI (ATBI). Green circle shows genes that were significantly altered in the comparison of Young TBI vs Aged TBI. A total of 193 genes were altered by TBI compared to Sham (Supplemental Table 4), with the majority (137) altered by TBI in both young and aged animals. For other divisions within the Venn, the genes within each group are designated as follows: # = C7, Ccl20, IL23a; *= Nox4, Ccl5; ¶ = Aire, Camp, Cxcl13, Ifna1, II5, Marco, Pdcd1; & = Defb1, Fcamr. [file Image_1.tiff]
