# Supplementary material for: Traumatic Brain Injury Induces cGAS Activation and Type I Interferon Signaling in Aged Mice
Source: Front Immunol. 2021 Aug 24;12:710608. doi: 10.3389/fimmu.2021.710608 (PMC8423402; doi:10.3389/fimmu.2021.710608)
Supplement: Supplementary file 2 [file Table_1.docx]

Barrett JP, Knoblach SM, Bhattacharya S, Gordish-Dressman H, Stoica BA and Loane DJ (2021) Traumatic Brain Injury Induces cGAS Activation and Type I Interferon Signaling in Aged Mice. *Front. Immunol.* 12:710608. doi: 10.3389/fimmu.2021.710608

**Supplementary Tables 1-6.**

| **Supplemental Table 1: Immune Transcripts Significantly Affected By TBI Alone in Young Adult and Aged Mice** | | | | | | | | | | | | | | |
| --- | --- | --- | --- | --- | --- | --- | --- | --- | --- | --- | --- | --- | --- | --- |
| **gene** | **model** | **N** | **model r2** | **injury coefficient** | **injury SE** | **unadj injury p-value** | **adj injury p-value** | **age coefficient** | **age SE** | **unadj age p-value** | **adj age p-value** |  | **Normality p-value - uninjured** | **Normality p-value - TBI** |
| **Cd14** | Linear regression | 23 | 0.9843 | 3.003 | 0.085 | 1.65E-19 | 9.28E-17 | 0.054 | 0.085 | 0.530 | 0.988 |  | 0.7152 | 0.6116 |
| **Socs3** | Linear regression | 23 | 0.9742 | 2.631 | 0.096 | 2.36E-17 | 1.32E-14 | -0.123 | 0.096 | 0.214 | 0.988 |  | 0.7089 | 0.5887 |
| **Cd44** | Linear regression | 23 | 0.9738 | 3.287 | 0.121 | 2.70E-17 | 1.51E-14 | -0.117 | 0.121 | 0.344 | 0.988 |  | 0.0968 | 0.5188 |
| **Ccl9** | Linear regression | 23 | 0.9723 | 2.733 | 0.103 | 4.89E-17 | 2.73E-14 | 0.066 | 0.103 | 0.530 | 0.988 |  | 0.3466 | 0.7332 |
| **Plaur** | Linear regression | 23 | 0.9711 | 2.555 | 0.099 | 8.49E-17 | 4.73E-14 | -0.448 | 0.099 | 2.15E-04 | 0.114 |  | 0.5377 | 0.3619 |
| **Ccl3** | Linear regression | 23 | 0.9692 | 3.285 | 0.133 | 1.79E-16 | 9.96E-14 | 0.399 | 0.133 | 0.007 | 0.988 |  | 0.9761 | 0.8764 |
| **Ccl2** | Linear regression | 23 | 0.9680 | 5.101 | 0.208 | 2.12E-16 | 1.18E-13 | 0.152 | 0.208 | 0.472 | 0.988 |  | 0.4120 | 0.6328 |
| **Lilrb4** | Linear regression | 23 | 0.9598 | 3.748 | 0.174 | 2.51E-15 | 1.39E-12 | 0.434 | 0.174 | 0.021 | 0.988 |  | 0.8269 | 0.8485 |
| **Ccl4** | Linear regression | 23 | 0.9591 | 3.040 | 0.140 | 2.38E-15 | 1.32E-12 | -0.281 | 0.140 | 0.060 | 0.988 |  | 0.8240 | 0.1126 |
| **Ccl12** | Linear regression | 23 | 0.9575 | 3.587 | 0.173 | 5.50E-15 | 3.03E-12 | 0.625 | 0.173 | 0.002 | 0.861 |  | 0.7305 | 0.7146 |
| **Il1rn** | Linear regression | 23 | 0.9558 | 3.971 | 0.191 | 5.14E-15 | 2.84E-12 | -0.132 | 0.191 | 0.497 | 0.988 |  | 0.0645 | 0.1493 |
| **Il13ra1** | Linear regression | 23 | 0.9487 | 1.236 | 0.064 | 2.30E-14 | 1.27E-11 | -0.034 | 0.064 | 0.602 | 0.988 |  | 0.6210 | 0.9811 |
| **Cxcl10** | Linear regression | 23 | 0.9467 | 3.886 | 0.209 | 4.42E-14 | 2.42E-11 | 0.503 | 0.209 | 0.026 | 0.988 |  | 0.9687 | 0.5219 |
| **Stat3** | Linear regression | 23 | 0.9465 | 1.058 | 0.057 | 4.34E-14 | 2.38E-11 | 0.115 | 0.057 | 0.056 | 0.988 |  | 0.8030 | 0.5554 |
| **Abcb1a** | Linear regression | 23 | 0.9410 | -1.085 | 0.064 | 2.19E-13 | 1.20E-10 | -0.285 | 0.064 | 2.25E-04 | 0.118 |  | 0.4269 | 0.1351 |
| **Nfkb2** | Linear regression | 23 | 0.9342 | 1.262 | 0.075 | 2.88E-13 | 1.57E-10 | -0.134 | 0.075 | 0.089 | 0.988 |  | 0.9953 | 0.6377 |
| **S100a8** | Linear regression | 23 | 0.9339 | 3.110 | 0.185 | 2.95E-13 | 1.61E-10 | -0.304 | 0.185 | 0.116 | 0.988 |  | 0.9705 | 0.3455 |
| **Arhgdib** | Linear regression | 23 | 0.9295 | 0.817 | 0.052 | 1.01E-12 | 5.42E-10 | -0.248 | 0.052 | 1.16E-04 | 0.062 |  | 0.0699 | 0.1109 |
| **Fcer1g** | Linear regression | 23 | 0.9292 | 0.851 | 0.053 | 6.86E-13 | 3.71E-10 | 0.079 | 0.053 | 0.154 | 0.988 |  | 0.1435 | 0.5619 |
| **Bcl3** | Linear regression | 23 | 0.9289 | 2.243 | 0.139 | 6.21E-13 | 3.38E-10 | 0.025 | 0.139 | 0.859 | 0.988 |  | 0.0777 | 0.5482 |
| **Lif** | Linear regression | 23 | 0.9286 | 3.229 | 0.200 | 6.41E-13 | 3.48E-10 | -0.007 | 0.200 | 0.972 | 0.988 |  | 0.4606 | 0.8405 |
| **Il6** | Linear regression | 23 | 0.9282 | 2.686 | 0.167 | 6.65E-13 | 3.60E-10 | -0.049 | 0.167 | 0.775 | 0.988 |  | 0.3489 | 0.5736 |
| **Fcgrt** | Linear regression | 23 | 0.9274 | -0.989 | 0.062 | 7.45E-13 | 4.02E-10 | 0.059 | 0.062 | 0.353 | 0.988 |  | 0.3756 | 0.3370 |
| **Cd244** | Linear regression | 23 | 0.9232 | 1.905 | 0.124 | 1.48E-12 | 7.98E-10 | 0.136 | 0.124 | 0.285 | 0.988 |  | 0.2956 | 0.5911 |
| **Clec4e** | Linear regression | 23 | 0.9217 | 2.656 | 0.173 | 1.58E-12 | 8.48E-10 | -0.122 | 0.173 | 0.490 | 0.988 |  | 0.3737 | 0.3190 |
| **Tlr2** | Linear regression | 23 | 0.9205 | 1.682 | 0.111 | 2.16E-12 | 1.16E-09 | 0.146 | 0.111 | 0.205 | 0.988 |  | 0.3752 | 0.5456 |
| **Phlpp1** | Linear regression | 23 | 0.9183 | -0.797 | 0.053 | 2.60E-12 | 1.39E-09 | -0.032 | 0.053 | 0.559 | 0.988 |  | 0.0018 | 0.5374 |
| **S100a9** | Linear regression | 23 | 0.9180 | 2.772 | 0.185 | 2.53E-12 | 1.35E-09 | -0.107 | 0.185 | 0.571 | 0.988 |  | 0.5176 | 0.3918 |
| **Msr1** | Linear regression | 23 | 0.9174 | 3.629 | 0.244 | 2.71E-12 | 1.45E-09 | -0.161 | 0.244 | 0.516 | 0.988 |  | 0.1126 | 0.6898 |
| **Cxcl3** | Linear regression | 23 | 0.9122 | 3.273 | 0.227 | 5.01E-12 | 2.66E-09 | -0.207 | 0.227 | 0.372 | 0.988 |  | 0.0198 | 0.2475 |
| **Fcgr2b** | Linear regression | 23 | 0.9098 | 1.067 | 0.076 | 7.54E-12 | 4.00E-09 | 0.086 | 0.076 | 0.269 | 0.988 |  | 0.0782 | 0.4207 |
| **Il1b** | Linear regression | 23 | 0.9085 | 2.760 | 0.197 | 8.30E-12 | 4.40E-09 | -0.399 | 0.197 | 0.056 | 0.988 |  | 0.3113 | 0.4872 |
| **Tgfbi** | Linear regression | 23 | 0.9055 | 1.870 | 0.135 | 1.07E-11 | 5.64E-09 | -0.164 | 0.135 | 0.240 | 0.988 |  | 0.0111 | 0.9445 |
| **Cxcl1** | Linear regression | 23 | 0.9030 | 3.265 | 0.239 | 1.37E-11 | 7.23E-09 | -0.227 | 0.239 | 0.355 | 0.988 |  | 0.6499 | 0.3205 |
| **Cd34** | Linear regression | 23 | 0.9010 | -1.256 | 0.093 | 1.67E-11 | 8.80E-09 | 0.036 | 0.093 | 0.701 | 0.988 |  | 0.9946 | 0.2448 |
| **C2** | Linear regression | 23 | 0.8973 | -1.686 | 0.128 | 2.44E-11 | 1.28E-08 | 0.131 | 0.128 | 0.316 | 0.988 |  | 0.9552 | 0.2791 |
| **Ncf4** | Linear regression | 23 | 0.8954 | 1.551 | 0.119 | 3.13E-11 | 1.64E-08 | 0.068 | 0.119 | 0.574 | 0.988 |  | 0.1839 | 0.2920 |
| **Runx1** | Linear regression | 23 | 0.8948 | 0.928 | 0.071 | 3.10E-11 | 1.63E-08 | -0.078 | 0.071 | 0.289 | 0.988 |  | 0.4856 | 0.1328 |
| **Frmpd4** | Linear regression | 23 | 0.8925 | -0.494 | 0.041 | 1.06E-10 | 5.48E-08 | -0.149 | 0.041 | 0.002 | 0.764 |  | 0.3872 | 0.2709 |
| **Il1r2** | Linear regression | 23 | 0.8912 | 2.094 | 0.164 | 4.52E-11 | 2.37E-08 | 0.059 | 0.164 | 0.725 | 0.988 |  | 0.4889 | 0.4892 |
| **App** | Linear regression | 23 | 0.8883 | -0.546 | 0.046 | 1.60E-10 | 8.24E-08 | -0.168 | 0.046 | 0.002 | 0.777 |  | 0.1514 | 0.3293 |
| **Ppbp** | Linear regression | 23 | 0.8862 | 3.305 | 0.265 | 6.96E-11 | 3.63E-08 | -0.338 | 0.265 | 0.217 | 0.988 |  | 0.5851 | 0.0916 |
| **Il18** | Linear regression | 23 | 0.8861 | -0.706 | 0.061 | 2.81E-10 | 1.43E-07 | -0.262 | 0.061 | 3.78E-04 | 0.196 |  | 0.0591 | 0.3606 |
| **Vtn** | Linear regression | 23 | 0.8847 | -0.967 | 0.080 | 1.11E-10 | 5.74E-08 | 0.241 | 0.080 | 0.007 | 0.988 |  | 0.9077 | 0.5447 |
| **Irgm1** | Linear regression | 23 | 0.8827 | 1.079 | 0.093 | 2.59E-10 | 1.32E-07 | 0.331 | 0.093 | 0.002 | 0.988 |  | 0.7688 | 0.4165 |
| **Ifi204** | Linear regression | 23 | 0.8823 | 2.064 | 0.176 | 2.11E-10 | 1.08E-07 | 0.540 | 0.176 | 0.006 | 0.988 |  | 0.0155 | 0.3687 |
| **Cybb** | Linear regression | 23 | 0.8796 | 1.341 | 0.111 | 1.25E-10 | 6.46E-08 | 0.040 | 0.111 | 0.722 | 0.988 |  | 0.6068 | 0.6253 |
| **Nfkbiz** | Linear regression | 23 | 0.8756 | 0.813 | 0.069 | 1.74E-10 | 8.92E-08 | 0.022 | 0.069 | 0.750 | 0.988 |  | 0.4684 | 0.9361 |
| **Tlr1** | Linear regression | 23 | 0.8755 | 1.307 | 0.110 | 1.73E-10 | 8.89E-08 | 0.015 | 0.110 | 0.896 | 0.988 |  | 0.5851 | 0.9662 |
| **Cxcr2** | Linear regression | 23 | 0.8746 | 2.437 | 0.207 | 1.96E-10 | 1.00E-07 | -0.347 | 0.207 | 0.110 | 0.988 |  | 0.8362 | 0.6131 |
| **Csf2rb** | Linear regression | 23 | 0.8729 | 1.614 | 0.141 | 3.00E-10 | 1.52E-07 | 0.263 | 0.141 | 0.076 | 0.988 |  | 0.8729 | 0.2312 |
| **Itga5** | Linear regression | 23 | 0.8702 | 1.749 | 0.152 | 2.72E-10 | 1.38E-07 | 0.070 | 0.152 | 0.649 | 0.988 |  | 0.0006 | 0.7759 |
| **Fcgr4** | Linear regression | 23 | 0.8690 | 2.732 | 0.237 | 2.84E-10 | 1.44E-07 | -0.006 | 0.237 | 0.981 | 0.988 |  | 0.1158 | 0.2339 |
| **Cdh5** | Linear regression | 23 | 0.8690 | 0.878 | 0.078 | 4.18E-10 | 2.10E-07 | -0.230 | 0.078 | 0.008 | 0.988 |  | 0.7417 | 0.8854 |
| **Alas1** | Linear regression | 23 | 0.8684 | -0.413 | 0.038 | 9.28E-10 | 4.61E-07 | 0.174 | 0.038 | 1.98E-04 | 0.105 |  | 0.8581 | 0.6577 |
| **Cradd** | Linear regression | 23 | 0.8674 | -0.612 | 0.054 | 3.18E-10 | 1.60E-07 | 0.011 | 0.054 | 0.846 | 0.988 |  | 0.1029 | 0.8011 |
| **Fcgr1** | Linear regression | 23 | 0.8649 | 1.115 | 0.100 | 4.65E-10 | 2.33E-07 | 0.120 | 0.100 | 0.244 | 0.988 |  | 0.1646 | 0.2229 |
| **Tnfrsf1b** | Linear regression | 23 | 0.8640 | 1.260 | 0.112 | 4.12E-10 | 2.07E-07 | -0.021 | 0.112 | 0.854 | 0.988 |  | 0.8668 | 0.4616 |
| **Cd86** | Linear regression | 23 | 0.8629 | 0.791 | 0.072 | 6.97E-10 | 3.48E-07 | 0.148 | 0.072 | 0.054 | 0.988 |  | 0.8951 | 0.5699 |
| **Ccl5** | Linear regression | 23 | 0.8618 | 1.810 | 0.182 | 3.42E-09 | 1.68E-06 | 0.838 | 0.182 | 1.70E-04 | 0.090 |  | 0.4045 | 0.4228 |
| **Sell** | Linear regression | 23 | 0.8615 | 2.614 | 0.239 | 6.98E-10 | 3.48E-07 | -0.653 | 0.239 | 0.013 | 0.988 |  | 0.5408 | 0.9551 |
| **Tbp** | Linear regression | 23 | 0.8602 | -0.429 | 0.044 | 4.80E-09 | 2.34E-06 | -0.213 | 0.044 | 1.01E-04 | 0.054 |  | 0.7438 | 0.3991 |
| **Blnk** | Linear regression | 23 | 0.8586 | 0.931 | 0.085 | 6.39E-10 | 3.19E-07 | 0.034 | 0.085 | 0.689 | 0.988 |  | 0.2954 | 0.7786 |
| **Cul9** | Linear regression | 23 | 0.8553 | -0.532 | 0.052 | 1.91E-09 | 9.39E-07 | 0.203 | 0.052 | 0.001 | 0.414 |  | 0.8891 | 0.2992 |
| **Il33** | Linear regression | 23 | 0.8490 | 1.219 | 0.115 | 1.18E-09 | 5.85E-07 | -0.095 | 0.115 | 0.420 | 0.988 |  | 0.0007 | 0.5766 |
| **Hprt** | Linear regression | 23 | 0.8483 | -0.400 | 0.043 | 8.73E-09 | 4.17E-06 | -0.187 | 0.043 | 2.81E-04 | 0.147 |  | 0.2734 | 0.5779 |
| **Irf7** | Linear regression | 23 | 0.8470 | 1.574 | 0.165 | 7.05E-09 | 3.40E-06 | 0.664 | 0.165 | 0.001 | 0.340 |  | 0.6497 | 0.4937 |
| **Mme** | Linear regression | 23 | 0.8461 | -1.374 | 0.131 | 1.42E-09 | 7.03E-07 | 0.049 | 0.131 | 0.712 | 0.988 |  | 0.5219 | 0.1605 |
| **Ptprc** | Linear regression | 23 | 0.8459 | 1.113 | 0.108 | 1.91E-09 | 9.39E-07 | 0.154 | 0.108 | 0.169 | 0.988 |  | 0.7607 | 0.5395 |
| **Ltbr** | Linear regression | 23 | 0.8433 | 0.760 | 0.074 | 2.13E-09 | 1.05E-06 | 0.090 | 0.074 | 0.239 | 0.988 |  | 0.9753 | 0.8202 |
| **Il19** | Linear regression | 23 | 0.8416 | 2.048 | 0.199 | 1.90E-09 | 9.39E-07 | -0.138 | 0.199 | 0.496 | 0.988 |  | 0.6687 | 0.8263 |
| **Ctsc** | Linear regression | 23 | 0.8389 | 1.019 | 0.104 | 4.40E-09 | 2.15E-06 | -0.340 | 0.104 | 0.004 | 0.988 |  | 0.9720 | 0.2103 |
| **Cd274** | Linear regression | 23 | 0.8354 | 1.157 | 0.123 | 9.32E-09 | 4.44E-06 | 0.403 | 0.123 | 0.004 | 0.988 |  | 0.7339 | 0.7166 |
| **Icam5** | Linear regression | 23 | 0.8350 | -0.714 | 0.076 | 9.10E-09 | 4.34E-06 | 0.308 | 0.076 | 0.001 | 0.319 |  | 0.9284 | 0.2006 |
| **Mapk1** | Linear regression | 23 | 0.8310 | -0.532 | 0.054 | 3.98E-09 | 1.95E-06 | 0.079 | 0.054 | 0.158 | 0.988 |  | 0.3728 | 0.0141 |
| **Tgfb3** | Linear regression | 23 | 0.8304 | -0.468 | 0.048 | 5.16E-09 | 2.50E-06 | 0.113 | 0.048 | 0.030 | 0.988 |  | 0.8892 | 0.7382 |
| **G6pdx** | Linear regression | 23 | 0.8288 | -0.295 | 0.030 | 5.45E-09 | 2.64E-06 | -0.041 | 0.030 | 0.199 | 0.988 |  | 0.5992 | 0.4380 |
| **Casp8** | Linear regression | 23 | 0.8254 | 0.750 | 0.077 | 5.10E-09 | 2.48E-06 | -0.057 | 0.077 | 0.469 | 0.988 |  | 0.8231 | 0.5759 |
| **Ptpn6** | Linear regression | 23 | 0.8236 | 0.781 | 0.081 | 6.25E-09 | 3.02E-06 | -0.124 | 0.081 | 0.143 | 0.988 |  | 0.8685 | 0.5713 |
| **Jak2** | Linear regression | 23 | 0.8195 | -0.387 | 0.044 | 2.40E-08 | 1.11E-05 | -0.138 | 0.044 | 0.005 | 0.988 |  | 0.8053 | 0.6092 |
| **Tyrobp** | Linear regression | 23 | 0.8190 | 0.475 | 0.050 | 7.38E-09 | 3.55E-06 | -0.003 | 0.050 | 0.951 | 0.988 |  | 0.6506 | 0.8977 |
| **Cdkn1a** | Linear regression | 23 | 0.8185 | 1.627 | 0.172 | 7.61E-09 | 3.66E-06 | 0.004 | 0.172 | 0.981 | 0.988 |  | 0.0086 | 0.0191 |
| **Il4ra** | Linear regression | 23 | 0.8172 | 0.977 | 0.103 | 8.17E-09 | 3.92E-06 | -0.005 | 0.103 | 0.964 | 0.988 |  | 0.9095 | 0.5978 |
| **Tnfrsf17** | Linear regression | 23 | 0.8171 | -1.278 | 0.143 | 2.09E-08 | 9.71E-06 | -0.390 | 0.143 | 0.013 | 0.988 |  | 0.9961 | 0.7876 |
| **H2_K1** | Linear regression | 23 | 0.8162 | 0.655 | 0.073 | 1.92E-08 | 8.98E-06 | 0.182 | 0.073 | 0.021 | 0.988 |  | 0.3230 | 0.4937 |
| **Trem1** | Linear regression | 23 | 0.8150 | 2.352 | 0.252 | 1.02E-08 | 4.86E-06 | -0.391 | 0.252 | 0.137 | 0.988 |  | 0.5864 | 0.6303 |
| **Bst2** | Linear regression | 23 | 0.8149 | 1.322 | 0.155 | 4.22E-08 | 1.91E-05 | 0.542 | 0.155 | 0.002 | 0.988 |  | 0.8177 | 0.4766 |
| **Csf1** | Linear regression | 23 | 0.8141 | 0.546 | 0.060 | 1.42E-08 | 6.67E-06 | 0.097 | 0.060 | 0.121 | 0.988 |  | 0.8843 | 0.3810 |
| **Pecam1** | Linear regression | 23 | 0.8104 | 0.636 | 0.069 | 1.33E-08 | 6.31E-06 | -0.112 | 0.069 | 0.123 | 0.988 |  | 0.7894 | 0.2150 |
| **Ahr** | Linear regression | 23 | 0.8087 | -0.503 | 0.058 | 2.96E-08 | 1.36E-05 | -0.144 | 0.058 | 0.022 | 0.988 |  | 0.2242 | 0.7323 |
| **Myd88** | Linear regression | 23 | 0.8077 | 0.939 | 0.103 | 1.37E-08 | 6.45E-06 | 0.004 | 0.103 | 0.966 | 0.988 |  | 0.0187 | 0.5161 |
| **Litaf** | Linear regression | 23 | 0.8077 | 0.662 | 0.073 | 1.45E-08 | 6.83E-06 | -0.096 | 0.073 | 0.203 | 0.988 |  | 0.0076 | 0.3425 |
| **Cd74** | Linear regression | 23 | 0.8075 | 1.258 | 0.137 | 1.36E-08 | 6.43E-06 | -0.086 | 0.137 | 0.538 | 0.988 |  | 0.3585 | 0.6646 |
| **Rorc** | Linear regression | 23 | 0.8038 | -0.884 | 0.098 | 1.87E-08 | 8.73E-06 | 0.150 | 0.098 | 0.143 | 0.988 |  | 0.2099 | 0.9640 |
| **Il1r1** | Linear regression | 23 | 0.8027 | 0.729 | 0.082 | 2.43E-08 | 1.13E-05 | -0.184 | 0.082 | 0.038 | 0.988 |  | 0.7020 | 0.5771 |
| **C8g** | Linear regression | 23 | 0.8021 | -0.737 | 0.083 | 2.45E-08 | 1.13E-05 | 0.180 | 0.083 | 0.044 | 0.988 |  | 0.9504 | 0.1398 |
| **Il17rb** | Linear regression | 23 | 0.8017 | -0.656 | 0.079 | 6.56E-08 | 2.95E-05 | -0.243 | 0.079 | 0.006 | 0.988 |  | 0.0544 | 0.4229 |
| **Tnf** | Linear regression | 23 | 0.8003 | 1.728 | 0.194 | 2.05E-08 | 9.56E-06 | 0.047 | 0.194 | 0.810 | 0.988 |  | 0.8525 | 0.5132 |
| **Atm** | Linear regression | 23 | 0.7966 | -0.328 | 0.042 | 1.42E-07 | 6.21E-05 | -0.151 | 0.042 | 0.002 | 0.830 |  | 0.0761 | 0.3726 |
| **Il2rg** | Linear regression | 23 | 0.7946 | 1.533 | 0.174 | 2.62E-08 | 1.21E-05 | -0.084 | 0.174 | 0.633 | 0.988 |  | 0.1313 | 0.6211 |
| **Ccl6** | Linear regression | 23 | 0.7918 | 1.588 | 0.184 | 3.59E-08 | 1.64E-05 | -0.312 | 0.184 | 0.106 | 0.988 |  | 0.0863 | 0.0003 |
| **Npc1** | Linear regression | 23 | 0.7916 | -0.499 | 0.057 | 3.03E-08 | 1.39E-05 | 0.014 | 0.057 | 0.811 | 0.988 |  | 0.1470 | 0.0369 |
| **Lilrb3** | Linear regression | 23 | 0.7916 | 1.439 | 0.165 | 3.03E-08 | 1.39E-05 | -0.037 | 0.165 | 0.825 | 0.988 |  | 0.6499 | 0.5752 |
| **Ets1** | Linear regression | 23 | 0.7906 | 0.562 | 0.075 | 2.95E-07 | 1.28E-04 | -0.349 | 0.075 | 1.44E-04 | 0.076 |  | 0.0156 | 0.7338 |
| **Entpd1** | Linear regression | 23 | 0.7895 | -0.612 | 0.083 | 3.99E-07 | 1.72E-04 | -0.348 | 0.083 | 4.44E-04 | 0.229 |  | 0.0674 | 0.7528 |
| **Tlr4** | Linear regression | 23 | 0.7890 | 0.647 | 0.075 | 3.74E-08 | 1.70E-05 | 0.038 | 0.075 | 0.617 | 0.988 |  | 0.0973 | 0.4208 |
| **Maf** | Linear regression | 23 | 0.7854 | -0.757 | 0.089 | 4.08E-08 | 1.85E-05 | 0.016 | 0.089 | 0.854 | 0.988 |  | 0.8087 | 0.1556 |
| **Cd36** | Linear regression | 23 | 0.7846 | 1.475 | 0.173 | 4.32E-08 | 1.95E-05 | 0.010 | 0.173 | 0.952 | 0.988 |  | 0.0599 | 0.7443 |
| **Cd48** | Linear regression | 23 | 0.7845 | 0.732 | 0.093 | 1.43E-07 | 6.24E-05 | 0.266 | 0.093 | 0.009 | 0.988 |  | 0.1864 | 0.6773 |
| **Ccr2** | Linear regression | 23 | 0.7799 | 2.102 | 0.254 | 6.81E-08 | 3.06E-05 | -0.479 | 0.254 | 0.074 | 0.988 |  | 0.8165 | 0.6090 |
| **Clec5a** | Linear regression | 23 | 0.7776 | 0.663 | 0.081 | 7.73E-08 | 3.46E-05 | 0.096 | 0.081 | 0.249 | 0.988 |  | 0.1295 | 0.4315 |
| **Masp1** | Linear regression | 23 | 0.7757 | -0.562 | 0.069 | 8.70E-08 | 3.89E-05 | -0.088 | 0.069 | 0.218 | 0.988 |  | 0.8073 | 0.6173 |
| **Stat5a** | Linear regression | 23 | 0.7680 | 0.779 | 0.096 | 9.75E-08 | 4.35E-05 | -0.116 | 0.096 | 0.240 | 0.988 |  | 0.4581 | 0.5749 |
| **Tgfb1** | Linear regression | 23 | 0.7667 | 1.056 | 0.131 | 9.79E-08 | 4.36E-05 | -0.116 | 0.131 | 0.386 | 0.988 |  | 0.0063 | 0.1591 |
| **Tmem173** | Linear regression | 23 | 0.7661 | 1.149 | 0.142 | 1.02E-07 | 4.54E-05 | -0.146 | 0.142 | 0.317 | 0.988 |  | 0.3297 | 0.7387 |
| **Mapk14** | Linear regression | 23 | 0.7646 | -0.389 | 0.048 | 1.07E-07 | 4.76E-05 | 0.044 | 0.048 | 0.378 | 0.988 |  | 0.9270 | 0.0525 |
| **Icam1** | Linear regression | 23 | 0.7638 | 0.858 | 0.107 | 1.13E-07 | 4.99E-05 | -0.106 | 0.107 | 0.332 | 0.988 |  | 0.8226 | 0.8582 |
| **Plau** | Linear regression | 23 | 0.7617 | 0.982 | 0.123 | 1.18E-07 | 5.20E-05 | -0.058 | 0.123 | 0.644 | 0.988 |  | 0.1293 | 0.9560 |
| **Syk** | Linear regression | 23 | 0.7615 | 0.657 | 0.087 | 2.65E-07 | 1.15E-04 | -0.249 | 0.087 | 0.009 | 0.988 |  | 0.6932 | 0.9901 |
| **Cd226** | Linear regression | 23 | 0.7594 | 2.017 | 0.255 | 1.38E-07 | 6.06E-05 | -0.267 | 0.255 | 0.307 | 0.988 |  | 0.0256 | 0.2150 |
| **Thy1** | Linear regression | 23 | 0.7589 | -0.474 | 0.060 | 1.57E-07 | 6.87E-05 | 0.093 | 0.060 | 0.139 | 0.988 |  | 0.4489 | 0.0012 |
| **Psmb5** | Linear regression | 23 | 0.7512 | -0.363 | 0.049 | 3.60E-07 | 1.55E-04 | 0.128 | 0.049 | 0.016 | 0.988 |  | 0.7733 | 0.1323 |
| **Bst1** | Linear regression | 23 | 0.7512 | 1.475 | 0.191 | 1.97E-07 | 8.61E-05 | 0.084 | 0.191 | 0.665 | 0.988 |  | 0.0134 | 0.1180 |
| **Ddx58** | Linear regression | 23 | 0.7497 | 0.781 | 0.112 | 9.52E-07 | 3.98E-04 | 0.346 | 0.112 | 0.006 | 0.988 |  | 0.4492 | 0.0924 |
| **Itgax** | Linear regression | 23 | 0.7467 | 0.737 | 0.100 | 4.00E-07 | 1.72E-04 | 0.180 | 0.100 | 0.087 | 0.988 |  | 0.6610 | 0.0865 |
| **Irak1** | Linear regression | 23 | 0.7423 | -0.310 | 0.047 | 1.99E-06 | 0.001 | -0.162 | 0.047 | 0.003 | 0.988 |  | 0.4425 | 0.5156 |
| **Cd81** | Linear regression | 23 | 0.7392 | -0.290 | 0.047 | 5.81E-06 | 0.002 | -0.196 | 0.047 | 0.001 | 0.268 |  | 0.3982 | 0.4400 |
| **Itgal** | Linear regression | 23 | 0.7382 | 1.013 | 0.135 | 3.07E-07 | 1.33E-04 | -0.020 | 0.135 | 0.884 | 0.988 |  | 0.9790 | 0.8258 |
| **Casp3** | Linear regression | 23 | 0.7372 | -0.437 | 0.069 | 3.38E-06 | 0.001 | -0.253 | 0.069 | 0.001 | 0.743 |  | 0.9574 | 0.9818 |
| **Psmd7** | Linear regression | 23 | 0.7370 | -0.351 | 0.048 | 4.07E-07 | 1.74E-04 | 0.079 | 0.048 | 0.111 | 0.988 |  | 0.8685 | 0.1104 |
| **C7** | Linear regression | 23 | 0.7362 | -0.883 | 0.123 | 5.76E-07 | 2.45E-04 | 0.287 | 0.123 | 0.030 | 0.988 |  | 0.3262 | 0.0955 |
| **Ifngr1** | Linear regression | 23 | 0.7326 | -0.240 | 0.038 | 3.96E-06 | 0.002 | -0.139 | 0.038 | 0.002 | 0.818 |  | 0.0143 | 0.6827 |
| **Ube2l3** | Linear regression | 23 | 0.7297 | -0.371 | 0.052 | 5.73E-07 | 2.44E-04 | -0.059 | 0.052 | 0.264 | 0.988 |  | 0.6552 | 0.0788 |
| **Ccl11** | Linear regression | 23 | 0.7295 | 1.304 | 0.179 | 4.78E-07 | 2.04E-04 | -0.223 | 0.179 | 0.228 | 0.988 |  | 0.9101 | 0.6710 |
| **Atg16l1** | Linear regression | 23 | 0.7287 | -0.455 | 0.065 | 7.81E-07 | 3.29E-04 | -0.109 | 0.065 | 0.106 | 0.988 |  | 0.9813 | 0.7031 |
| **Bcl2** | Linear regression | 23 | 0.7238 | -0.475 | 0.066 | 5.78E-07 | 2.45E-04 | -0.032 | 0.066 | 0.633 | 0.988 |  | 0.7804 | 0.3498 |
| **Spn** | Linear regression | 23 | 0.7229 | 1.033 | 0.149 | 9.57E-07 | 3.99E-04 | -0.340 | 0.149 | 0.033 | 0.988 |  | 0.6279 | 0.0084 |
| **Cxcr4** | Linear regression | 23 | 0.7199 | 1.018 | 0.142 | 6.07E-07 | 2.57E-04 | -0.043 | 0.142 | 0.766 | 0.988 |  | 0.5704 | 0.5554 |
| **Tnfaip3** | Linear regression | 23 | 0.7194 | 0.518 | 0.075 | 9.86E-07 | 4.10E-04 | -0.157 | 0.075 | 0.049 | 0.988 |  | 0.5679 | 0.2038 |
| **Oaz1** | Linear regression | 23 | 0.7188 | -0.400 | 0.057 | 7.34E-07 | 3.10E-04 | 0.076 | 0.057 | 0.195 | 0.988 |  | 0.7929 | 0.9824 |
| **Ebi3** | Linear regression | 23 | 0.7181 | -0.401 | 0.061 | 2.08E-06 | 0.001 | 0.187 | 0.061 | 0.006 | 0.988 |  | 0.0449 | 0.6726 |
| **Phlpp2** | Linear regression | 23 | 0.7172 | -0.518 | 0.074 | 8.19E-07 | 3.44E-04 | -0.064 | 0.074 | 0.399 | 0.988 |  | 0.5112 | 0.9467 |
| **H2_Ab1** | Linear regression | 23 | 0.7102 | 1.075 | 0.156 | 1.04E-06 | 4.32E-04 | 0.128 | 0.156 | 0.421 | 0.988 |  | 0.3692 | 0.7048 |
| **Traf6** | Linear regression | 23 | 0.7095 | -0.290 | 0.042 | 9.21E-07 | 3.86E-04 | -0.010 | 0.042 | 0.815 | 0.988 |  | 0.1224 | 0.5758 |
| **Hlx** | Linear regression | 23 | 0.7095 | 1.018 | 0.152 | 1.58E-06 | 0.001 | 0.250 | 0.152 | 0.114 | 0.988 |  | 0.8358 | 0.6724 |
| **Ikbkap** | Linear regression | 23 | 0.7079 | -0.363 | 0.058 | 3.84E-06 | 0.002 | 0.189 | 0.058 | 0.004 | 0.988 |  | 0.4611 | 0.1489 |
| **Ptafr** | Linear regression | 23 | 0.7064 | 0.756 | 0.109 | 1.05E-06 | 4.33E-04 | 0.037 | 0.109 | 0.737 | 0.988 |  | 0.6332 | 0.8826 |
| **Zeb1** | Linear regression | 23 | 0.7032 | -0.376 | 0.055 | 1.21E-06 | 4.98E-04 | 0.061 | 0.055 | 0.281 | 0.988 |  | 0.1407 | 0.1474 |
| **Tollip** | Linear regression | 23 | 0.6993 | -0.364 | 0.054 | 1.43E-06 | 0.001 | 0.067 | 0.054 | 0.228 | 0.988 |  | 0.9329 | 0.9561 |
| **Irf5** | Linear regression | 23 | 0.6991 | 0.509 | 0.075 | 1.30E-06 | 0.001 | 0.010 | 0.075 | 0.894 | 0.988 |  | 0.6802 | 0.1327 |
| **Gapdh** | Linear regression | 23 | 0.6985 | -0.317 | 0.049 | 2.65E-06 | 0.001 | -0.090 | 0.049 | 0.082 | 0.988 |  | 0.3519 | 0.2682 |
| **Sdha** | Linear regression | 23 | 0.6980 | -0.363 | 0.054 | 1.50E-06 | 0.001 | 0.067 | 0.054 | 0.228 | 0.988 |  | 0.4225 | 0.9110 |
| **Stat2** | Linear regression | 23 | 0.6976 | 0.847 | 0.134 | 3.76E-06 | 0.001 | 0.302 | 0.134 | 0.036 | 0.988 |  | 0.2526 | 0.0249 |
| **Slamf7** | Linear regression | 23 | 0.6961 | 1.300 | 0.192 | 1.42E-06 | 0.001 | -0.124 | 0.192 | 0.528 | 0.988 |  | 0.6306 | 0.5006 |
| **Ikbkb** | Linear regression | 23 | 0.6877 | -0.311 | 0.061 | 5.76E-05 | 0.021 | -0.247 | 0.061 | 0.001 | 0.327 |  | 0.2076 | 0.7322 |
| **Ly86** | Linear regression | 23 | 0.6864 | 0.397 | 0.067 | 8.58E-06 | 0.003 | 0.180 | 0.067 | 0.015 | 0.988 |  | 0.9905 | 0.0400 |
| **Cd9** | Linear regression | 23 | 0.6842 | 0.656 | 0.103 | 3.28E-06 | 0.001 | -0.203 | 0.103 | 0.063 | 0.988 |  | 0.0213 | 0.1663 |
| **Jak3** | Linear regression | 23 | 0.6841 | 0.474 | 0.072 | 2.15E-06 | 0.001 | 0.015 | 0.072 | 0.839 | 0.988 |  | 1.0000 | 0.4061 |
| **Il18rap** | Linear regression | 23 | 0.6790 | 1.306 | 0.211 | 4.80E-06 | 0.002 | 0.363 | 0.211 | 0.101 | 0.988 |  | 0.6152 | 0.5752 |
| **Fyn** | Linear regression | 23 | 0.6774 | -0.385 | 0.063 | 5.45E-06 | 0.002 | -0.114 | 0.063 | 0.084 | 0.988 |  | 0.6141 | 0.5304 |
| **Nfkbia** | Linear regression | 23 | 0.6751 | 0.478 | 0.077 | 4.52E-06 | 0.002 | 0.109 | 0.077 | 0.170 | 0.988 |  | 0.3508 | 0.6577 |
| **Stat5b** | Linear regression | 23 | 0.6737 | -0.378 | 0.061 | 4.32E-06 | 0.002 | -0.076 | 0.061 | 0.223 | 0.988 |  | 0.1707 | 0.3847 |
| **Irak4** | Linear regression | 23 | 0.6692 | 0.422 | 0.066 | 3.32E-06 | 0.001 | -0.029 | 0.066 | 0.670 | 0.988 |  | 0.9520 | 0.9745 |
| **Cfh** | Linear regression | 23 | 0.6691 | -0.613 | 0.102 | 7.23E-06 | 0.003 | -0.187 | 0.102 | 0.082 | 0.988 |  | 0.2266 | 0.8040 |
| **Irf1** | Linear regression | 23 | 0.6670 | 0.436 | 0.071 | 4.89E-06 | 0.002 | -0.116 | 0.071 | 0.115 | 0.988 |  | 0.9682 | 0.4631 |
| **Cd1d1** | Linear regression | 23 | 0.6644 | 1.056 | 0.175 | 6.83E-06 | 0.003 | -0.365 | 0.175 | 0.051 | 0.988 |  | 0.3911 | 0.3864 |
| **Casp1** | Linear regression | 23 | 0.6641 | -0.457 | 0.097 | 1.30E-04 | 0.044 | 0.421 | 0.097 | 3.03E-04 | 0.158 |  | 0.7599 | 0.9187 |
| **Card9** | Linear regression | 23 | 0.6621 | 0.412 | 0.078 | 3.30E-05 | 0.012 | 0.237 | 0.078 | 0.006 | 0.988 |  | 0.9278 | 0.6067 |
| **Ptgs2** | Linear regression | 23 | 0.6591 | 1.464 | 0.235 | 4.51E-06 | 0.002 | -0.034 | 0.235 | 0.885 | 0.988 |  | 0.1620 | 0.3513 |
| **H2_Eb1** | Linear regression | 23 | 0.6586 | 1.003 | 0.164 | 5.50E-06 | 0.002 | -0.213 | 0.164 | 0.209 | 0.988 |  | 0.1325 | 0.5734 |
| **Tlr3** | Linear regression | 23 | 0.6582 | -0.352 | 0.059 | 8.12E-06 | 0.003 | -0.089 | 0.059 | 0.149 | 0.988 |  | 0.4692 | 0.0018 |
| **Ifnar1** | Linear regression | 23 | 0.6505 | -0.279 | 0.054 | 5.05E-05 | 0.018 | -0.166 | 0.054 | 0.006 | 0.988 |  | 0.1083 | 0.6097 |
| **Ifitm1** | Linear regression | 23 | 0.6496 | 0.833 | 0.141 | 9.17E-06 | 0.003 | -0.255 | 0.141 | 0.087 | 0.988 |  | 0.1348 | 0.6500 |
| **Tcf4** | Linear regression | 23 | 0.6477 | -0.431 | 0.072 | 6.86E-06 | 0.003 | -0.030 | 0.072 | 0.684 | 0.988 |  | 0.8616 | 0.8132 |
| **Tirap** | Linear regression | 23 | 0.6474 | 0.400 | 0.078 | 5.28E-05 | 0.019 | 0.236 | 0.078 | 0.007 | 0.988 |  | 0.9981 | 0.1629 |
| **Cxcl12** | Linear regression | 23 | 0.6441 | -0.523 | 0.093 | 1.62E-05 | 0.006 | -0.171 | 0.093 | 0.080 | 0.988 |  | 0.0161 | 0.0996 |
| **Ifi35** | Linear regression | 23 | 0.6408 | 0.579 | 0.116 | 7.03E-05 | 0.025 | 0.354 | 0.116 | 0.006 | 0.988 |  | 0.7223 | 0.3346 |
| **Muc1** | Linear regression | 23 | 0.6391 | 0.693 | 0.117 | 8.65E-06 | 0.003 | -0.103 | 0.117 | 0.387 | 0.988 |  | 0.2719 | 0.3248 |
| **Mr1** | Linear regression | 23 | 0.6363 | -0.601 | 0.102 | 9.12E-06 | 0.003 | 0.076 | 0.102 | 0.463 | 0.988 |  | 0.5622 | 0.3417 |
| **Clu** | Linear regression | 23 | 0.6345 | -0.354 | 0.060 | 9.84E-06 | 0.004 | -0.021 | 0.060 | 0.734 | 0.988 |  | 0.0143 | 0.5733 |
| **Kit** | Linear regression | 23 | 0.6340 | -0.495 | 0.084 | 9.47E-06 | 0.004 | 0.048 | 0.084 | 0.571 | 0.988 |  | 0.4335 | 0.8432 |
| **Cd19** | Linear regression | 23 | 0.6340 | 0.998 | 0.176 | 1.48E-05 | 0.006 | -0.318 | 0.176 | 0.085 | 0.988 |  | 0.0319 | 0.5957 |
| **Src** | Linear regression | 23 | 0.6327 | -0.352 | 0.068 | 4.56E-05 | 0.017 | 0.203 | 0.068 | 0.007 | 0.988 |  | 0.6267 | 0.1768 |
| **Trp53** | Linear regression | 23 | 0.6318 | 0.343 | 0.063 | 2.67E-05 | 0.010 | -0.157 | 0.063 | 0.022 | 0.988 |  | 0.9788 | 0.6768 |
| **Tlr9** | Linear regression | 23 | 0.6219 | -0.600 | 0.113 | 3.31E-05 | 0.012 | 0.269 | 0.113 | 0.027 | 0.988 |  | 0.5360 | 0.1142 |
| **Tnfsf10** | Linear regression | 23 | 0.6203 | -0.816 | 0.143 | 1.37E-05 | 0.005 | 0.002 | 0.143 | 0.988 | 0.988 |  | 0.9279 | 0.3433 |
| **Lcp2** | Linear regression | 23 | 0.6052 | -0.503 | 0.100 | 6.22E-05 | 0.022 | 0.251 | 0.100 | 0.021 | 0.988 |  | 0.8831 | 0.0923 |
| **Itga2b** | Linear regression | 23 | 0.5945 | 1.131 | 0.230 | 8.45E-05 | 0.029 | 0.474 | 0.230 | 0.053 | 0.988 |  | 0.8329 | 0.8906 |
| **Relb** | Linear regression | 23 | 0.5940 | 0.527 | 0.103 | 5.24E-05 | 0.019 | 0.156 | 0.103 | 0.145 | 0.988 |  | 0.6172 | 0.9175 |
| **C1qb** | Linear regression | 23 | 0.5939 | 0.191 | 0.040 | 1.03E-04 | 0.036 | 0.088 | 0.040 | 0.038 | 0.988 |  | 0.8339 | 0.7673 |
| **Tnfsf12** | Linear regression | 23 | 0.5935 | -0.392 | 0.073 | 3.01E-05 | 0.011 | -0.031 | 0.073 | 0.673 | 0.988 |  | 0.0396 | 0.5404 |
| **Ccr7** | Linear regression | 23 | 0.5905 | 0.710 | 0.137 | 4.72E-05 | 0.017 | -0.235 | 0.137 | 0.103 | 0.988 |  | 0.8172 | 0.6253 |
| **Il16** | Linear regression | 23 | 0.5905 | -0.675 | 0.128 | 3.62E-05 | 0.013 | -0.094 | 0.128 | 0.469 | 0.988 |  | 0.9149 | 0.9129 |
| **Pdgfrb** | Linear regression | 23 | 0.5893 | -0.350 | 0.074 | 1.20E-04 | 0.041 | -0.165 | 0.074 | 0.036 | 0.988 |  | 0.2600 | 0.4261 |
| **Tgfbr1** | Linear regression | 23 | 0.5758 | -0.425 | 0.082 | 4.79E-05 | 0.017 | -0.042 | 0.082 | 0.618 | 0.988 |  | 0.1513 | 0.2971 |
| **Batf** | Linear regression | 23 | 0.5725 | 0.541 | 0.105 | 4.93E-05 | 0.018 | 0.034 | 0.105 | 0.747 | 0.988 |  | 0.5884 | 0.4044 |
| **Smad3** | Linear regression | 23 | 0.5717 | -0.379 | 0.073 | 4.81E-05 | 0.017 | 0.041 | 0.073 | 0.583 | 0.988 |  | 0.2271 | 0.8690 |
| **Cd40** | Linear regression | 23 | 0.5704 | 0.495 | 0.101 | 8.24E-05 | 0.029 | -0.176 | 0.101 | 0.095 | 0.988 |  | 0.5886 | 0.5395 |
| **Nfil3** | Linear regression | 23 | 0.5704 | 0.558 | 0.109 | 5.45E-05 | 0.020 | -0.105 | 0.109 | 0.348 | 0.988 |  | 0.5083 | 0.7204 |
| **Cd7** | Linear regression | 23 | 0.5638 | 0.751 | 0.149 | 6.40E-05 | 0.023 | 0.075 | 0.149 | 0.619 | 0.988 |  | 0.9309 | 0.1072 |
| **Csf3r** | Linear regression | 23 | 0.5619 | 0.837 | 0.167 | 6.92E-05 | 0.024 | 0.100 | 0.167 | 0.557 | 0.988 |  | 0.9210 | 0.7642 |
| **Nfatc3** | Linear regression | 23 | 0.5598 | -0.242 | 0.051 | 1.27E-04 | 0.043 | -0.078 | 0.051 | 0.144 | 0.988 |  | 0.9961 | 0.0275 |
| **Ifngr2** | Linear regression | 23 | 0.5593 | -0.250 | 0.051 | 7.97E-05 | 0.028 | -0.040 | 0.051 | 0.441 | 0.988 |  | 0.0142 | 0.7426 |
| **Cd163** | Linear regression | 23 | 0.5565 | -0.914 | 0.183 | 6.72E-05 | 0.024 | 0.049 | 0.183 | 0.792 | 0.988 |  | 0.8959 | 0.0856 |
| **Cd209g** | Linear regression | 23 | 0.5564 | -0.997 | 0.199 | 6.76E-05 | 0.024 | 0.070 | 0.199 | 0.728 | 0.988 |  | 0.9864 | 0.1123 |
| **Tnfsf14** | Linear regression | 23 | 0.5421 | 1.184 | 0.245 | 1.02E-04 | 0.035 | -0.203 | 0.245 | 0.418 | 0.988 |  | 0.2411 | 0.8061 |
| **H2_Aa** | Linear regression | 23 | 0.5413 | 0.882 | 0.185 | 1.21E-04 | 0.041 | -0.224 | 0.185 | 0.241 | 0.988 |  | 0.9547 | 0.6036 |
| **H2_Q10** | Linear regression | 23 | 0.5347 | 1.246 | 0.264 | 1.34E-04 | 0.046 | -0.292 | 0.264 | 0.282 | 0.988 |  | 0.6067 | 0.3242 |
| **C3** | Linear regression | 23 | 0.5330 | 1.541 | 0.323 | 1.17E-04 | 0.040 | 0.010 | 0.323 | 0.976 | 0.988 |  | 0.8076 | 0.7186 |
| **Ncam1** | Linear regression | 23 | 0.5308 | -0.282 | 0.060 | 1.40E-04 | 0.047 | -0.034 | 0.060 | 0.574 | 0.988 |  | 0.0588 | 0.8302 |
| R2 represents the overall coefficient for the regression model that TBI and age affect transcript expression (higher value=greater effect). It is the amount of variability in expression that is accounted for by the combination of TBI and age. The injury coefficient reflects the difference in expression between subjects with TBI vs shams, while accounting for age. It is the prediction of expression given that all mice were the same age. Positive (increased) or negative (decreased) values indicate expression in mice with TBI compared to shams. The age coefficient reflects the difference in expression between aged or young mice, while accounting for TBI. Positive (increased) or negative (decreased) values indicate expression in aged mice compared to young mice. Note the scale is a log scale. SE=Standard error of the coefficient. Unadjusted injury or age values reflect the raw p-value testing the null hypotheses that the injury or age coefficients are 0, respectively. Adjusted p-values represent the raw p-values with Benjamini-Hochberg correction for multiple comparisons. Normality columns show the normality of individual gene expression values in Sham/uninjured or TBI mice, respectively, where a p-value <0.05 indicates a departure from normality (highlighted in blue). The lower the p-value, the greater the departure from normality, signifying that regression results for these genes should be interpreted with caution. | | | | | | | | | | | | | | |
|  |  |  |  |  |  |  |  |  |  |  |  |  |  |  |
|  |  |  |  |  |  |  |  |  |  |  |  |  |  |  |
|  |  |  |  |  |  |  |  |  |  |  |  |  |  |  |
|  |  |  |  |  |  |  |  |  |  |  |  |  |  |  |

| **Supplemental Table 2: Immune Transcripts Significantly Affected By Age Alone in Young Adult and Aged Mice** | | | | | | | | | | | | | | | |
| --- | --- | --- | --- | --- | --- | --- | --- | --- | --- | --- | --- | --- | --- | --- | --- |
| **gene** | **model** | **N** | **model r2** | **injury coefficient** | **injury SE** | **unadj injury p-value** | **adj injury p-value** | **age coefficient** | **age SE** | **unadj age p-value** | **adj age p-value** |  | **Normality p-value - uninjured** | **Normality p-value - TBI** |  |
| **Nfkb1** | Linear regression | 23 | 0.8821 | 0.035 | 0.062 | 0.585 | 0.999 | 0.758 | 0.062 | 1.04E-10 | 5.85E-08 |  | 0.2808 | 0.0234 |  |
| **Il1rap** | Linear regression | 23 | 0.8718 | 0.104 | 0.043 | 0.026 | 0.999 | -0.498 | 0.043 | 2.84E-10 | 1.59E-07 |  | 0.1434 | 0.3000 |  |
| **Il6st** | Linear regression | 23 | 0.8384 | 0.140 | 0.060 | 0.031 | 0.999 | -0.604 | 0.060 | 3.11E-09 | 1.73E-06 |  | 0.7229 | 0.0420 |  |
| **Adal** | Linear regression | 23 | 0.8133 | -0.283 | 0.077 | 0.002 | 0.437 | -0.650 | 0.077 | 5.36E-08 | 2.97E-05 |  | 0.5362 | 0.5378 |  |
| **Traf2** | Linear regression | 23 | 0.7794 | -0.119 | 0.076 | 0.134 | 0.999 | 0.636 | 0.076 | 6.29E-08 | 3.49E-05 |  | 0.0425 | 0.1780 |  |
| **C1qa** | Linear regression | 23 | 0.7772 | 0.089 | 0.034 | 0.015 | 0.999 | 0.261 | 0.034 | 1.75E-07 | 9.66E-05 |  | 0.1590 | 0.7750 |  |
| **Eef1g** | Linear regression | 23 | 0.7689 | -0.072 | 0.057 | 0.224 | 0.999 | 0.463 | 0.057 | 9.44E-08 | 5.22E-05 |  | 0.5924 | 0.0251 |  |
| **Traf3** | Linear regression | 23 | 0.7370 | -0.354 | 0.080 | 2.55E-04 | 0.083 | -0.465 | 0.080 | 1.07E-05 | 0.006 |  | 0.9285 | 0.4775 |  |
| **Defb1** | Linear regression | 23 | 0.7368 | -0.572 | 0.152 | 0.001 | 0.350 | 1.006 | 0.152 | 1.87E-06 | 0.001 |  | 0.6712 | 0.2421 |  |
| **Il10** | Linear regression | 23 | 0.6381 | -0.444 | 0.156 | 0.010 | 0.999 | -0.789 | 0.156 | 5.83E-05 | 0.032 |  | 0.4685 | 0.7338 |  |
| **Vcam1** | Linear regression | 23 | 0.5834 | -0.163 | 0.100 | 0.118 | 0.999 | -0.493 | 0.100 | 7.63E-05 | 0.041 |  | 0.7333 | 0.1776 |  |
| **Cxcl9** | Linear regression | 23 | 0.5607 | -0.114 | 0.153 | 0.464 | 0.999 | -0.760 | 0.153 | 7.58E-05 | 0.041 |  | 0.0487 | 0.7069 |  |
| R2 represents the overall coefficient for the regression model that TBI and age affect transcript expression (higher value=greater effect). It is the amount of variability in expression that is accounted for by the combination of TBI and age. The injury coefficient reflects the difference in expression between subjects with TBI vs shams, while accounting for age. It is the prediction of expression given that all mice were the same age. Positive (increased) or negative (decreased) values indicate expression in mice with TBI compared to shams. The age coefficient reflects the difference in expression between aged or young mice, while accounting for TBI. Positive (increased) or negative (decreased) values indicate expression in aged mice compared to young mice. Note the scale is a log scale. SE=Standard error of the coefficient. Unadjusted injury or age values reflect the raw p-value testing the null hypotheses that the injury or age coefficients are 0, respectively. Unadjusted p-value <0.05 is highlighted in red. Adjusted p-values represent the raw p-values with Benjamini-Hochberg correction for multiple comparisons. Adjusted p-value <0.05 is highlighted in red. Normality columns show the normality of individual gene expression values in Sham/uninjured or TBI mice, respectively, where a p-value <0.05 indicates a departure from normality (highlighted in blue). The lower the p-value, the greater the departure from normality, signifying that regression results for these genes should be interpreted with caution. | | | | | | | | | | | | | | | |
|  |  |  |  |  |  |  |  |  |  |  |  |  |  |  |  |
|  |  |  |  |  |  |  |  |  |  |  |  |  |  |  |  |
|  |  |  |  |  |  |  |  |  |  |  |  |  |  |  |  |
|  |  |  |  |  |  |  |  |  |  |  |  |  |  |  |  |

| **Supplemental Table 3: Immune Transcripts Significantly Affected By TBI and AGE in Young Adult and Aged Mice** | | | | | | | | | | | | | | | |
| --- | --- | --- | --- | --- | --- | --- | --- | --- | --- | --- | --- | --- | --- | --- | --- |
| **gene** | **model** | **N** | **model r2** | **injury coefficient** | **injury SE** | **unadj injury p-value** | **adj injury p-value** | **age coefficient** | **age SE** | **unadj age p-value** | **adj age p-value** |  | **Normality p-value - uninjured** | **Normality p-value - TBI** |  |
| **Cebpb** | Linear regression | 23 | 0.9075 | 0.916 | 0.075 | 1.04E-10 | 5.38E-08 | 0.478 | 0.075 | 3.37E-06 | 0.002 |  | 0.8955 | 0.7657 |  |
| **Csf1r** | Linear regression | 23 | 0.9061 | -0.530 | 0.043 | 7.02E-11 | 3.65E-08 | -0.238 | 0.043 | 1.80E-05 | 0.010 |  | 0.2482 | 0.3161 |  |
| **Ccr5** | Linear regression | 23 | 0.8986 | 0.806 | 0.065 | 6.75E-11 | 3.52E-08 | -0.335 | 0.065 | 4.42E-05 | 0.024 |  | 0.2693 | 0.2326 |  |
| **Tap1** | Linear regression | 23 | 0.8948 | 1.143 | 0.097 | 1.77E-10 | 9.05E-08 | 0.481 | 0.097 | 7.31E-05 | 0.039 |  | 0.3109 | 0.5571 |  |
| **Itgb2** | Linear regression | 23 | 0.8937 | 0.979 | 0.114 | 3.86E-08 | 1.75E-05 | 1.063 | 0.114 | 1.02E-08 | 5.67E-06 |  | 0.0939 | 0.2573 |  |
| **Tnfrsf11a** | Linear regression | 23 | 0.8920 | -0.279 | 0.053 | 4.20E-05 | 0.015 | -0.614 | 0.053 | 2.88E-10 | 1.61E-07 |  | 0.2004 | 0.0578 |  |
| **Ppia** | Linear regression | 23 | 0.8830 | -0.370 | 0.051 | 5.43E-07 | 2.32E-04 | -0.491 | 0.051 | 6.30E-09 | 3.51E-06 |  | 0.1953 | 0.0539 |  |
| **C4a** | Linear regression | 23 | 0.8812 | 1.212 | 0.134 | 1.59E-08 | 7.48E-06 | 1.031 | 0.134 | 2.04E-07 | 1.13E-04 |  | 0.0418 | 0.1990 |  |
| **Cx3cl1** | Linear regression | 23 | 0.8357 | -0.692 | 0.079 | 2.59E-08 | 1.19E-05 | 0.418 | 0.079 | 3.31E-05 | 0.018 |  | 0.8144 | 0.8578 |  |
| **Ccl7** | Linear regression | 23 | 0.7883 | 1.406 | 0.216 | 2.39E-06 | 0.001 | -1.285 | 0.216 | 8.06E-06 | 0.004 |  | 0.0054 | 0.0277 |  |
| **Tbk1** | Linear regression | 23 | 0.7857 | -0.347 | 0.058 | 8.27E-06 | 0.003 | 0.376 | 0.058 | 2.84E-06 | 0.002 |  | 0.4688 | 0.4337 |  |
| **Ctss** | Linear regression | 23 | 0.7579 | -0.251 | 0.048 | 3.93E-05 | 0.014 | 0.294 | 0.048 | 5.12E-06 | 0.003 |  | 0.2841 | 0.1697 |  |
| **Ilf3** | Linear regression | 23 | 0.7522 | -0.303 | 0.048 | 3.93E-06 | 0.002 | 0.236 | 0.048 | 8.82E-05 | 0.047 |  | 0.6323 | 0.7938 |  |
| **Psmb7** | Linear regression | 23 | 0.7125 | -0.316 | 0.064 | 7.44E-05 | 0.026 | 0.331 | 0.064 | 4.23E-05 | 0.023 |  | 0.3965 | 0.7225 |  |
| R2 represents the overall coefficient for the regression model that TBI and age affect transcript expression (higher value=greater effect). It is the amount of variability in expression that is accounted for by the combination of TBI and age. The injury coefficient reflects the difference in expression between subjects with TBI vs shams, while accounting for age. It is the prediction of expression given that all mice were the same age. Positive (increased) or negative (decreased) values indicate expression in mice with TBI compared to shams. The age coefficient reflects the difference in expression between aged or young mice, while accounting for TBI. Positive (increased) or negative (decreased) values indicate expression in aged mice compared to young mice. Note the scale is a log scale. SE=Standard error of the coefficient. Unadjusted injury or age values reflect the raw p-value testing the null hypotheses that the injury or age coefficients are 0, respectively. Unadjusted p-value <0.05 is highlighted in red. Adjusted p-values represent the raw p-values with Benjamini-Hochberg correction for multiple comparisons. Adjusted p-value <0.05 is highlighted in red. Normality columns show the normality of individual gene expression values in Sham/uninjured or TBI mice, respectively, where a p-value <0.05 indicates a departure from normality (highlighted in blue). The lower the p-value, the greater the departure from normality, signifying that regression results for these genes should be interpreted with caution. | | | | | | | | | | | | | | | |
|  |  |  |  |  |  |  |  |  |  |  |  |  |  |  |  |
|  |  |  |  |  |  |  |  |  |  |  |  |  |  |  |  |
|  |  |  |  |  |  |  |  |  |  |  |  |  |  |  |  |
|  |  |  |  |  |  |  |  |  |  |  |  |  |  |  |  |

| **Supplemental Table 4. Significant Fold change and Adjusted p values- TBI vs Sham** | | | | | | | |
| --- | --- | --- | --- | --- | --- | --- | --- |
| Significantly changed expression was defined as adjusted p<0.05 and log fold change (positive or negative) greater than 1.5. 0=value so low that it is computationally 0 | | | | | | | |
| **Young Sham vs Young TBI** | | | | **Aged Sham vs Aged TBI** | | | |
| **Gene** | **Log Fold Change** | ***p* value** | **Adjusted *p* (qvalue)** | **Gene** | **Log Fold Change** | ***p* value** | **Adjusted *p* (qvalue)** |
| **Arhgdib** | 1.776175944 | 0 | 0 | **Aire** | 2.370976964 | 0.02655795 | 0.049244737 |
| **Batf** | 4.099976825 | 1.18E-07 | 3.71E-07 | **Batf** | 2.606001524 | 6.98E-08 | 2.29E-07 |
| **Bcl3** | 5.836746043 | 0 | 0 | **Bcl3** | 5.023725365 | 0 | 0 |
| **Blnk** | 1.783298101 | 0 | 0 | **Blnk** | 1.733883745 | 0 | 0 |
| **Bst1** | 24.97471347 | 5.16E-12 | 2.20E-11 | **Bst1** | 3.566674373 | 3.68E-08 | 1.24E-07 |
| **Bst2** | 1.919536596 | 4.27E-11 | 1.67E-10 | **Bst2** | 2.974163163 | 0 | 0 |
| **C2** | -3.47064147 | 6.66E-16 | 3.54E-15 | **C2** | -2.87091871 | 0 | 0 |
| **C3** | 3.41909297 | 0.00052979 | 0.00122276 | **C3** | 1.513217067 | 6.32E-11 | 2.79E-10 |
| **C4a** | 2.768023883 | 0 | 0 | **C4a** | 1.602049398 | 7.82E-11 | 3.40E-10 |
| **C7** | -2.00656686 | 6.28E-05 | 0.00015768 | **C8b** | 1.571941523 | 0.00180812 | 0.004086955 |
| **Casp8** | 1.604910073 | 0 | 0 | **Camp** | 19.38006744 | 0.0008925 | 0.002095269 |
| **Ccl11** | 27.47062928 | 0 | 0 | **Casp8** | 1.554223541 | 0 | 0 |
| **Ccl12** | 6.133869428 | 0 | 0 | **Ccl11** | 6.853320078 | 4.47E-13 | 2.26E-12 |
| **Ccl2** | 29.85755079 | 0 | 0 | **Ccl12** | 6.228017452 | 0 | 0 |
| **Ccl20** | 40.43269418 | 2.29E-10 | 8.57E-10 | **Ccl2** | 9.000277943 | 0 | 0 |
| **Ccl22** | 2.47633633 | 1.56E-08 | 5.25E-08 | **Ccl22** | 1.551965485 | 0.00010394 | 0.000268174 |
| **Ccl24** | 36.06041753 | 1.54E-06 | 4.38E-06 | **Ccl24** | 24.68891257 | 2.92E-09 | 1.07E-08 |
| **Ccl3** | 6.846064638 | 0 | 0 | **Ccl3** | 5.249716739 | 0 | 0 |
| **Ccl4** | 7.744278789 | 0 | 0 | **Ccl4** | 11.02211136 | 0 | 0 |
| **Ccl5** | 28.42144307 | 0 | 0 | **Ccl5** | 4.881753571 | 0 | 0 |
| **Ccl6** | 3.774351193 | 1.96E-12 | 8.66E-12 | **Ccl6** | 3.374963777 | 8.29E-11 | 3.57E-10 |
| **Ccl8** | 22.96536083 | 0.0046033 | 0.00943073 | **Ccl7** | 4.075680563 | 0 | 0 |
| **Ccl9** | 4.636919388 | 0 | 0 | **Ccl8** | 3.677228545 | 0.00923802 | 0.018176967 |
| **Ccr2** | 5.851951104 | 1.74E-14 | 8.51E-14 | **Ccl9** | 4.536421123 | 0 | 0 |
| **Ccr5** | 1.766314364 | 0 | 0 | **Ccr10** | 21.35505167 | 0.00354845 | 0.007702384 |
| **Ccr7** | 23.14748026 | 1.37E-14 | 6.85E-14 | **Ccr2** | 7.817678089 | 0 | 0 |
| **Ccrl2** | 2.177810702 | 6.90E-08 | 2.19E-07 | **Ccr5** | 1.683285799 | 0 | 0 |
| **Cd109** | 2.232568555 | 1.04E-06 | 3.01E-06 | **Ccr7** | 3.947355838 | 3.09E-07 | 9.67E-07 |
| **Cd14** | 5.175517988 | 0 | 0 | **Ccrl2** | 1.741774804 | 9.34E-07 | 2.82E-06 |
| **Cd19** | 24.23919496 | 8.65E-09 | 3.01E-08 | **Cd109** | 1.634108739 | 0.00016067 | 0.000401317 |
| **Cd1d1** | 2.93937697 | 2.22E-16 | 1.19E-15 | **Cd14** | 4.712074162 | 0 | 0 |
| **Cd2** | 30.97469465 | 5.64E-06 | 1.55E-05 | **Cd163** | -1.5358464 | 0.00155639 | 0.003547262 |
| **Cd22** | 3.298476009 | 2.57E-07 | 7.76E-07 | **Cd19** | 156.0218219 | 1.00E-07 | 3.27E-07 |
| **Cd226** | 26.75837219 | 1.97E-13 | 9.29E-13 | **Cd1d1** | 2.146014571 | 5.91E-10 | 2.34E-09 |
| **Cd244** | 191.0920073 | 0 | 0 | **Cd2** | 40.99584855 | 0.00011061 | 0.000284064 |
| **Cd274** | 2.160015873 | 2.22E-16 | 1.19E-15 | **Cd22** | 2.203336123 | 1.48E-06 | 4.41E-06 |
| **Cd34** | -1.68365907 | 0 | 0 | **Cd226** | 27.19688504 | 4.86E-12 | 2.33E-11 |
| **Cd36** | 4.169243652 | 1.83E-14 | 8.87E-14 | **Cd244** | 25.16143842 | 0 | 0 |
| **Cd40** | 1.724732737 | 5.91E-07 | 1.75E-06 | **Cd247** | 1.626195683 | 0.00884538 | 0.017530509 |
| **Cd44** | 5.533818787 | 0 | 0 | **Cd274** | 2.448453666 | 0 | 0 |
| **Cd48** | 2.258258875 | 0 | 0 | **Cd36** | 3.098294872 | 3.44E-11 | 1.57E-10 |
| **Cd53** | 1.805726839 | 2.45E-08 | 8.12E-08 | **Cd40** | 2.393594582 | 8.48E-10 | 3.22E-09 |
| **Cd7** | 1.660564705 | 5.28E-08 | 1.71E-07 | **Cd44** | 5.099325371 | 0 | 0 |
| **Cd74** | 2.561231168 | 0 | 0 | **Cd48** | 1.50574632 | 2.13E-11 | 9.98E-11 |
| **Cd79b** | 4.868531543 | 5.17E-06 | 1.43E-05 | **Cd53** | 2.164411589 | 1.54E-07 | 4.98E-07 |
| **Cd80** | 1.521408636 | 1.23E-08 | 4.15E-08 | **Cd7** | 1.901821354 | 1.40E-10 | 5.87E-10 |
| **Cd86** | 1.82615891 | 0 | 0 | **Cd74** | 2.039627979 | 1.22E-15 | 7.11E-15 |
| **Cdh5** | 1.759051822 | 0 | 0 | **Cd79b** | 3.376245733 | 8.56E-06 | 2.40E-05 |
| **Cdkn1a** | 2.799050806 | 1.55E-15 | 8.10E-15 | **Cd80** | 1.727004542 | 4.67E-10 | 1.87E-09 |
| **Cebpb** | 1.857926571 | 0 | 0 | **Cd86** | 1.541580718 | 0 | 0 |
| **Cfb** | 27.01036925 | 1.84E-05 | 4.84E-05 | **Cdh5** | 1.680235647 | 0 | 0 |
| **Ciita** | 28.63872886 | 5.99E-10 | 2.17E-09 | **Cdkn1a** | 2.609299074 | 1.65E-14 | 9.05E-14 |
| **Clec4e** | 26.99420848 | 0 | 0 | **Cebpb** | 1.629688784 | 0 | 0 |
| **Csf2rb** | 4.340711128 | 0 | 0 | **Cfb** | 84.48213308 | 4.01E-05 | 0.000107981 |
| **Csf3r** | 2.190773023 | 6.54E-09 | 2.31E-08 | **Ciita** | 24.83550655 | 4.81E-07 | 1.49E-06 |
| **Ctsc** | 1.841284122 | 0 | 0 | **Clec4e** | 45.42491064 | 0 | 0 |
| **Cxcl1** | 105.4911111 | 0 | 0 | **Clec5a** | 1.57221474 | 0 | 0 |
| **Cxcl10** | 27.94091937 | 0 | 0 | **Csf2rb** | 3.168686156 | 0 | 0 |
| **Cxcl11** | 20.43090003 | 0.01821002 | 0.03422982 | **Csf3r** | 1.66482807 | 2.46E-06 | 7.17E-06 |
| **Cxcl3** | 57.65594682 | 0 | 0 | **Ctsc** | 1.938567719 | 0 | 0 |
| **Cxcr2** | 70.4301321 | 0 | 0 | **Cxcl1** | 25.2855936 | 0 | 0 |
| **Cxcr4** | 1.977969606 | 2.10E-09 | 7.52E-09 | **Cxcl10** | 7.059566551 | 0 | 0 |
| **Cxcr5** | 25.57313676 | 0.0081305 | 0.0161723 | **Cxcl13** | 28.5481254 | 3.77E-10 | 1.51E-09 |
| **Cybb** | 2.645717895 | 0 | 0 | **Cxcl3** | 30.43411546 | 0 | 0 |
| **Eomes** | 28.20648652 | 1.40E-09 | 5.04E-09 | **Cxcl9** | 2.896448586 | 0.00399041 | 0.00862748 |
| **Fas** | 1.544362888 | 7.51E-12 | 3.19E-11 | **Cxcr2** | 26.97495107 | 0 | 0 |
| **Fcer1g** | 1.792232304 | 0 | 0 | **Cxcr4** | 2.118972935 | 4.51E-11 | 2.02E-10 |
| **Fcgr1** | 1.984495712 | 0 | 0 | **Cybb** | 3.05983731 | 0 | 0 |
| **Fcgr2b** | 2.084385233 | 0 | 0 | **Ddx58** | 2.034099592 | 0 | 0 |
| **Fcgr4** | 5.770917893 | 0 | 0 | **Eomes** | 22.31792593 | 1.28E-08 | 4.39E-08 |
| **Gpr183** | 1.729024472 | 3.19E-10 | 1.19E-09 | **Fcer1g** | 1.630485035 | 0 | 0 |
| **H2-Aa** | 1.987325687 | 4.40E-08 | 1.45E-07 | **Fcgr1** | 2.261586376 | 0 | 0 |
| **H2-Ab1** | 2.493874888 | 3.01E-14 | 1.44E-13 | **Fcgr2b** | 1.886922627 | 0 | 0 |
| **H2-Eb1** | 2.386268966 | 4.19E-10 | 1.54E-09 | **Fcgr4** | 6.92340585 | 0 | 0 |
| **H2-Ob** | 2.059149671 | 0.00024866 | 0.00058881 | **H2-Aa** | 1.746502299 | 5.87E-07 | 1.79E-06 |
| **H2-Q10** | 4.2812462 | 1.78E-08 | 5.93E-08 | **H2-Ab1** | 1.951539416 | 2.72E-10 | 1.12E-09 |
| **Hcst** | 3.029381684 | 0.0002089 | 0.00050117 | **H2-Eb1** | 2.195992235 | 3.46E-09 | 1.25E-08 |
| **Hlx** | 3.241961263 | 2.21E-13 | 1.03E-12 | **H2-Q10** | 28.58424033 | 9.73E-13 | 4.79E-12 |
| **Icam1** | 2.560588433 | 0 | 0 | **Hlx** | 2.575968078 | 2.42E-13 | 1.25E-12 |
| **Ifi204** | 26.23235158 | 0 | 0 | **Icam1** | 1.544648898 | 8.46E-10 | 3.22E-09 |
| **Ifitm1** | 1.955346168 | 1.46E-10 | 5.58E-10 | **Ifi204** | 6.290010384 | 0 | 0 |
| **Il13ra1** | 2.271703057 | 0 | 0 | **Ifi35** | 1.712897059 | 0 | 0 |
| **Il17b** | 2.779851751 | 2.35E-07 | 7.14E-07 | **Ifitm1** | 1.60650406 | 4.96E-08 | 1.65E-07 |
| **Il18rap** | 25.36778304 | 8.99E-07 | 2.62E-06 | **Ifna1** | 708.2117749 | 0.00755753 | 0.015204949 |
| **Il19** | 39.68208849 | 0 | 0 | **Ikbke** | 1.7715905 | 5.63E-08 | 1.86E-07 |
| **Il1b** | 4.935565635 | 0 | 0 | **Ikzf3** | 26.46090814 | 0.01093916 | 0.021294369 |
| **Il1r1** | 1.70063335 | 0 | 0 | **Il13ra1** | 2.199492823 | 0 | 0 |
| **Il1r2** | 5.662354367 | 0 | 0 | **Il17b** | 1.646582918 | 0.00090732 | 0.002111941 |
| **Il1rl1** | 2.106980698 | 0.00238008 | 0.00516628 | **Il18rap** | 26.72864365 | 5.30E-09 | 1.91E-08 |
| **Il1rn** | 73.97786418 | 0 | 0 | **Il19** | 27.42277743 | 0 | 0 |
| **Il21r** | 27.52337837 | 0.00186534 | 0.00409774 | **Il1a** | 3.190337849 | 3.60E-06 | 1.04E-05 |
| **Il23a** | 25.61943769 | 8.33E-07 | 2.44E-06 | **Il1b** | 6.351508164 | 0 | 0 |
| **Il27ra** | 2.948793656 | 0.00022641 | 0.00053845 | **Il1r2** | 5.558841521 | 0 | 0 |
| **Il2rb** | 1.609845107 | 1.49E-05 | 3.99E-05 | **Il1rl1** | 1.945637783 | 0.00263045 | 0.005755419 |
| **Il2rg** | 4.520218142 | 0 | 0 | **Il1rn** | 31.36555823 | 0 | 0 |
| **Il33** | 2.243052545 | 0 | 0 | **Il21r** | 43.16075501 | 0.00021961 | 0.000533885 |
| **Il4ra** | 2.094669121 | 0 | 0 | **Il22** | 27.0201699 | 0.00017397 | 0.000430593 |
| **Il6** | 24.22894297 | 0 | 0 | **Il27ra** | 22.04156231 | 5.02E-05 | 0.000133926 |
| **Il7r** | 2.053800194 | 3.20E-06 | 8.98E-06 | **Il2rg** | 3.538917435 | 0 | 0 |
| **Irf7** | 3.028775652 | 0 | 0 | **Il33** | 2.084984734 | 0 | 0 |
| **Irgm1** | 1.642503866 | 0 | 0 | **Il4ra** | 1.571109304 | 0 | 0 |
| **Itga2b** | 24.35921751 | 8.74E-09 | 3.03E-08 | **Il5** | 66.33638219 | 0.00427031 | 0.009124451 |
| **Itga5** | 3.439816791 | 0 | 0 | **Il6** | 6.98462755 | 0 | 0 |
| **Itgal** | 4.592575841 | 1.67E-15 | 8.59E-15 | **Il7r** | 3.059986277 | 3.74E-08 | 1.26E-07 |
| **Itgax** | 4.410005168 | 4.61E-14 | 2.19E-13 | **Irf7** | 3.487331665 | 0 | 0 |
| **Itgb2** | 2.353555712 | 1.11E-16 | 6.07E-16 | **Irgm1** | 2.279745755 | 0 | 0 |
| **Klrd1** | 129.0271459 | 4.07E-06 | 1.14E-05 | **Itga2b** | 4.87026899 | 3.97E-06 | 1.14E-05 |
| **Klrk1** | 20.35942827 | 0.00993203 | 0.01954251 | **Itga5** | 2.906083864 | 0 | 0 |
| **Lif** | 6.530803656 | 0 | 0 | **Itgal** | 2.821712872 | 6.62E-10 | 2.60E-09 |
| **Lilrb3** | 24.27891397 | 0 | 0 | **Itgax** | 2.207221103 | 8.08E-09 | 2.85E-08 |
| **Lilrb4** | 9.112316997 | 0 | 0 | **Itgb2** | 1.772480402 | 4.69E-14 | 2.49E-13 |
| **Lta** | 21.82484362 | 0.0021931 | 0.00477938 | **Klrd1** | 25.6425546 | 2.70E-05 | 7.46E-05 |
| **Ltb4r1** | 25.64290588 | 1.11E-16 | 6.07E-16 | **Klrk1** | 16.28354567 | 0.01884335 | 0.03566544 |
| **Ltbr** | 1.616105931 | 0 | 0 | **Lif** | 5.924065763 | 0 | 0 |
| **Map4k1** | 16.41164199 | 0.00347052 | 0.0072457 | **Lilra6** | 39.98670476 | 0.02577659 | 0.04812217 |
| **Masp2** | 2.794625967 | 0.00060824 | 0.00139792 | **Lilrb3** | 5.693421396 | 2.09E-12 | 1.02E-11 |
| **Ms4a1** | 2.941928323 | 5.14E-08 | 1.68E-07 | **Lilrb4** | 5.737900193 | 0 | 0 |
| **Msr1** | 141.1585773 | 0 | 0 | **Map4k1** | 1.504563404 | 0.00426428 | 0.009124451 |
| **Muc1** | 2.556739418 | 2.51E-11 | 1.02E-10 | **Marco** | 25.23972078 | 7.70E-10 | 2.97E-09 |
| **Myd88** | 1.95715835 | 0 | 0 | **Masp2** | 1.801764303 | 0.00588721 | 0.012198125 |
| **Ncf4** | 3.308808239 | 0 | 0 | **Mme** | -1.88610208 | 7.31E-10 | 2.84E-09 |
| **Nfkb2** | 2.472690358 | 0 | 0 | **Ms4a1** | 2.502345185 | 3.21E-06 | 9.29E-06 |
| **Nfkbiz** | 1.772281101 | 0 | 0 | **Msr1** | 26.17468159 | 0 | 0 |
| **Nod2** | 1.524493384 | 0.00252502 | 0.00543775 | **Muc1** | 1.728939829 | 1.59E-06 | 4.74E-06 |
| **Nox4** | 31.99585928 | 1.11E-16 | 6.07E-16 | **Myd88** | 1.616913383 | 0 | 0 |
| **Pax5** | 24.9909527 | 5.82E-05 | 0.00014744 | **Ncf4** | 3.076175128 | 0 | 0 |
| **Pdcd1lg2** | 26.59060777 | 1.33E-05 | 3.60E-05 | **Nfkb2** | 2.257073477 | 0 | 0 |
| **Plau** | 1.899197267 | 0 | 0 | **Nfkbiz** | 1.517836104 | 0 | 0 |
| **Plaur** | 4.207282638 | 0 | 0 | **Nox4** | 24.3865361 | 3.79E-05 | 0.000103211 |
| **Ppbp** | 8.219323171 | 0 | 0 | **Pax5** | 22.55100702 | 9.67E-05 | 0.000251855 |
| **Ptafr** | 2.094966668 | 8.74E-12 | 3.65E-11 | **Pdcd1** | 2.635255525 | 0.00112193 | 0.002589427 |
| **Ptger4** | 3.263757947 | 1.02E-08 | 3.48E-08 | **Pdcd1lg2** | 25.17726059 | 7.46E-09 | 2.65E-08 |
| **Ptgs2** | 2.795465278 | 2.20E-11 | 8.99E-11 | **Plau** | 1.724963616 | 0 | 0 |
| **Ptpn22** | 1.57469231 | 1.65E-06 | 4.65E-06 | **Plaur** | 4.414417361 | 0 | 0 |
| **Ptpn6** | 1.613609508 | 0 | 0 | **Ppbp** | 6.937067748 | 0 | 0 |
| **Ptprc** | 2.42962714 | 0 | 0 | **Ptafr** | 1.663468081 | 8.66E-09 | 3.04E-08 |
| **Runx1** | 1.991643062 | 0 | 0 | **Ptger4** | 2.899624839 | 7.21E-06 | 2.04E-05 |
| **S100a8** | 5.009864841 | 0 | 0 | **Ptgs2** | 2.866602588 | 2.37E-12 | 1.15E-11 |
| **S100a9** | 4.62496888 | 0 | 0 | **Ptpn6** | 1.627007201 | 0 | 0 |
| **Sele** | 2.144924076 | 0.00282994 | 0.00599628 | **Ptprc** | 2.049660344 | 2.22E-16 | 1.33E-15 |
| **Sell** | 30.78485293 | 0 | 0 | **Runx1** | 1.850302196 | 0 | 0 |
| **Slamf1** | 38.94257499 | 0.00101835 | 0.00232098 | **S100a8** | 5.120914588 | 0 | 0 |
| **Slamf7** | 27.30952518 | 4.75E-12 | 2.04E-11 | **S100a9** | 4.67763032 | 0 | 0 |
| **Socs1** | 1.611997633 | 1.15E-08 | 3.89E-08 | **Sele** | 3.665022505 | 0.00293269 | 0.006391166 |
| **Socs3** | 4.933630138 | 0 | 0 | **Sell** | 41.19757737 | 0 | 0 |
| **Spn** | 24.75950427 | 3.40E-12 | 1.49E-11 | **Slamf1** | 25.00102498 | 7.46E-05 | 0.000196266 |
| **Stat3** | 1.969663591 | 0 | 0 | **Slamf7** | 35.71694103 | 2.67E-13 | 1.37E-12 |
| **Stat5a** | 1.931055966 | 0 | 0 | **Socs1** | 1.622196413 | 1.77E-09 | 6.59E-09 |
| **Tap1** | 1.928681493 | 0 | 0 | **Socs3** | 4.485728193 | 0 | 0 |
| **Tgfb1** | 2.14137221 | 0 | 0 | **Spn** | 142.3623726 | 1.59E-10 | 6.61E-10 |
| **Tgfbi** | 3.11091185 | 0 | 0 | **Stat1** | 1.553304102 | 2.39E-14 | 1.29E-13 |
| **Tlr1** | 3.823254436 | 0 | 0 | **Stat2** | 2.08722577 | 2.22E-16 | 1.33E-15 |
| **Tlr2** | 4.816828209 | 0 | 0 | **Stat3** | 1.839663961 | 0 | 0 |
| **Tlr4** | 1.670445119 | 1.11E-16 | 6.07E-16 | **Syk** | 1.56405481 | 8.22E-15 | 4.54E-14 |
| **Tlr8** | 4.790184788 | 2.15E-07 | 6.65E-07 | **Tap1** | 2.450642494 | 0 | 0 |
| **Tmem173** | 18.25577611 | 0 | 0 | **Tgfb1** | 1.958249773 | 0 | 0 |
| **Tnf** | 48.40707488 | 0 | 0 | **Tgfbi** | 3.377687722 | 0 | 0 |
| **Tnfrsf13c** | 20.88305441 | 0.00710893 | 0.01434902 | **Tlr1** | 2.751811502 | 0 | 0 |
| **Tnfrsf17** | -38.1240394 | 0.00039023 | 0.00091221 | **Tlr2** | 3.341493434 | 0 | 0 |
| **Tnfrsf1b** | 2.520085277 | 0 | 0 | **Tlr5** | 3.674825184 | 0.00853089 | 0.017030653 |
| **Tnfsf11** | 27.80473878 | 0.00671962 | 0.01366406 | **Tmem173** | 4.375615824 | 3.33E-16 | 1.98E-15 |
| **Tnfsf14** | 77.091025 | 3.62E-11 | 1.43E-10 | **Tnf** | 26.82045794 | 0 | 0 |
| **Traf1** | 24.14723133 | 3.10E-13 | 1.44E-12 | **Tnfrsf13c** | 3.067295574 | 0.00013903 | 0.000352071 |
| **Trem1** | 81.60629601 | 0 | 0 | **Tnfrsf17** | -23.3615847 | 0.00186089 | 0.004188924 |
| **Tslp** | 2.688287518 | 2.88E-09 | 1.02E-08 | **Tnfrsf1b** | 2.395690484 | 0 | 0 |
| **Zap70** | 2.33313503 | 1.70E-05 | 4.49E-05 | **Tnfrsf9** | 22.82926664 | 0.00952381 | 0.018672133 |
|  |  |  |  | **Tnfsf11** | 25.92780842 | 0.00020263 | 0.000494809 |
|  |  |  |  | **Tnfsf14** | 25.63933345 | 2.97E-09 | 1.08E-08 |
|  |  |  |  | **Traf1** | 23.23125006 | 1.56E-07 | 5.01E-07 |
|  |  |  |  | **Trem1** | 28.08326537 | 0 | 0 |
|  |  |  |  | **Tslp** | 3.338050758 | 1.74E-09 | 6.51E-09 |

| **Supplemental Table 5 - Individual z-scores and genes that contribute to each canonical pathway in Ingenuity Pathway Analysis.** | | | | | | | | | | |  |
| --- | --- | --- | --- | --- | --- | --- | --- | --- | --- | --- | --- |
| **Canonical Pathways** | **z-score** | **-log(p-value)** | **Transcripts Significantly Altered by TBI Only** | | | | | | | **Ratio** | |
| Cardiac Hypertrophy Signalling (Enhanced) | 3.051 | 10.6 | ATP,CSF2RB,CXCR2,CYBB,IL13RA1,IL18,IL1B,IL1R2,IL6,ITGA5,LIF,NFKB2,STAT3,TNFRSF1B | | | | | | | 0.0275 | |
| TREM1 Signalling | 2.714 | 16.3 | CCL2,CD86,FCGR2B,IL18,IL1B,IL6,ITGA5,NFKB2,STAT3,TLR1,TLR2 | | | | |  |  | 0.145 | |
| Hepatic Fibrosis Signalling Pathway | 2.714 | 8.49 | CCL2,CCL5,CYBB,IL18,IL1B,IL1R2,IL1RN,ITGA5,NFKB2,STAT3,TNFRSF1B | | | | | |  | 0.0284 | |
| Osteoarthritis Pathway | 2.646 | 9.59 | ATP,CXCR2,IL1B,IL1R2,ITGA5,NFKB2,S100A8,S100A9,TLR2,TNFRSF1B | | | | |  |  | 0.0441 | |
| Tumour Microenvironment Pathway | 2.646 | 6.37 | CCL2,CD44,IL1B,IL6,ITGA5,NFKB2,STAT3 | | |  |  |  |  | 0.0378 | |
| Role of NFAT in Regulation of the Immune Response | 2.646 | 6.34 | BLNK,CD86,FCER1G,FCGR1A,FCGR2B,FCGR3A/FCGR3B,NFKB2 | | | | |  |  | 0.0374 | |
| Dendritic Cell Maturation | 2.53 | 13.4 | CD86,FCER1G,FCGR1A,FCGR2B,FCGR3A/FCGR3B,IL18,IL1B,IL1RN,IL6,NFKB2,TLR2,TNFRSF1B | | | | | | | 0.0649 | |
| Neuroinflammation Signalling Pathway | 2.496 | 12 | APP,ATP,CCL2,CCL5,CD86,CXCL10,CYBB,IL18,IL1B,IL6,NFKB2,TLR1,TLR2 | | | | | |  | 0.0413 | |
| Th17 Activation Pathway | 2.449 | 8.44 | FCER1G,IL1B,IL6,NFKB2,RUNX1,SOCS3,STAT3 | | |  |  |  |  | 0.0753 | |
| Role of Pattern Recognition Receptors in Recognition of Bacteria and Viruses | 2.449 | 8.3 | CCL5,IL18,IL1B,IL6,LIF,NFKB2,TLR1,TLR2 | | |  |  |  |  | 0.0519 | |
| Role of MAPK Signalling in Inhibiting the Pathogenesis of Influenza | 2.449 | 7.35 | CCL2,CCL5,CXCL10,IL1B,IL6,NFKB2 | |  |  |  |  |  | 0.0769 | |
| Production of Nitric Oxide and Reactive Oxygen Species in Macrophages | 2.449 | 5.01 | CYBB,NCF4,NFKB2,S100A8,TLR2,TNFRSF1B | | |  |  |  |  | 0.0308 | |
| Role of Hypercytokinemia/  hyperchemokinemia in the Pathogenesis of Influenza | 2.333 | 12 | CCL2,CCL4,CCL5,CXCL10,IL18,IL1B,IL1RN,IL6,NFKB2 | | | |  |  |  | 0.103 | |
| NF-κB Signalling | 2.333 | 9.17 | FCER1G,IL18,IL1B,IL1R2,IL1RN,NFKB2,TLR1,TLR2,TNFRSF1B | | | | |  |  | 0.0503 | |
| Differential Regulation of Cytokine Production in Macrophages and T Helper Cells by IL-17A and IL-17F | 2.236 | 9.14 | CCL2,CCL4,CCL5,IL1B,IL6 |  |  |  |  |  |  | 0.278 | |
| Role of IL-17F in Allergic Inflammatory Airway Diseases | 2.236 | 8.95 | CCL2,CCL4,CXCL10,IL1B,IL6,NFKB2 | |  |  |  |  |  | 0.14 | |
| LPS/IL-1 Mediated Inhibition of RXR Function | 2.236 | 6.95 | ABCB1,ALAS1,CD14,IL18,IL1B,IL1R2,IL1RN,TNFRSF1B | | | |  |  |  | 0.0348 | |
| LXR/RXR Activation | -2.714 | 15.4 | CCL2,CD14,IL18,IL1B,IL1R2,IL1RN,IL6,MSR1,NFKB2,S100A8,TNFRSF1B,VTN | | | | | |  | 0.0938 | |

| **Supplemental Table 6: Individual Interactions Schematized in Figure 5** | | |
| --- | --- | --- |
| Gene 1=Gene predicted to interact with Gene 2 | | |
| Gene 2=Input gene, the gene with significantly altered expression in the dataset | | |
| Weight=strength of prediction derived from the GeneMania Network "Predicted" | | |
|  | | |
| **Gene 1** | **Gene 2** | **Weight** |
| Tlr4 | Tlr3 | 0.054393895 |
| Cxcl10 | Ccl2 | 0.04373107 |
| Tlr2 | Tlr4 | 0.1605174 |
| Irf5 | Stat2 | 0.013843727 |
| C3 | Ifitm1 | 0.03416237 |
| Ifnar1 | Stat2 | 0.014757074 |
| Stat3 | Ets1 | 0.005963056 |
| Stat3 | Tap1 | 0.004769119 |
| Stat3 | Stat2 | 0.007903281 |
| Stat3 | Ifnar1 | 0.002893989 |
| Stat3 | Ifi35 | 0.012985047 |
| Ccl5 | Ccl2 | 0.06814622 |
| Ccl5 | Cxcl10 | 0.06772775 |
| Irf1 | Stat2 | 0.019158458 |
| Irf1 | Stat3 | 0.007228962 |
| Tbk1 | Relb | 0.011719565 |
| Nmi | Stat3 | 0.004912406 |
| Nmi | Irf1 | 0.012484674 |
| Stat1 | Ets1 | 0.003552562 |
| Stat1 | Stat2 | 0.011114387 |
| Stat1 | Ifnar1 | 0.006501165 |
| Stat1 | Ifi35 | 0.014683902 |
| Stat1 | Stat3 | 0.005772638 |
| Irf9 | Stat2 | 0.027722787 |
| Irf9 | Ifnar1 | 0.030447423 |
| Irf9 | Stat3 | 0.005933086 |
| Irf9 | Irf1 | 0.0481956 |
| Irf9 | Nmi | 0.018065473 |
| Irf9 | Stat1 | 0.011831965 |
| Cxcl9 | Ccl2 | 0.04657938 |
| Cxcl9 | Cxcl10 | 0.19077943 |
| Cxcl9 | Ccl5 | 0.07383681 |
| Ifit1 | Tap1 | 0.01443784 |
| Ifit2 | Ifit1 | 0.19678421 |
| Tapbp | Tap1 | 0.051635087 |
| Eif2ak2 | Tlr4 | 0.014312194 |
| Eif2ak2 | Irf1 | 0.010474802 |
| Eif2ak2 | Nmi | 0.007720531 |
| Eif2ak2 | Irf9 | 0.015157633 |
| Tmem173 | Ddx58 | 0.05724077 |
| Irf7 | Tmem173 | 0.046942934 |
| Irf7 | Cxcl10 | 0.06124338 |
| Irf7 | Irf5 | 0.0352152 |
| Stat3 | Ifnar1 | 0.042885844 |
| Ccl5 | Irf7 | 0.033954173 |
| Ccl5 | Relb | 0.048673443 |
| Irf1 | Ddx58 | 0.010659025 |
| Irf1 | Tlr3 | 0.01771027 |
| Irf1 | Tap1 | 0.051179424 |
| Irf1 | Cxcl10 | 0.026322063 |
| Irf1 | Irf5 | 0.015135297 |
| Irf1 | Irf7 | 0.008741426 |
| Irf1 | Stat3 | 0.006382387 |
| Irf1 | Ccl5 | 0.014593314 |
| Tbk1 | Ddx58 | 0.028959222 |
| Tbk1 | Tmem173 | 0.05481513 |
| Tbk1 | Irf5 | 0.04112069 |
| Tbk1 | Irf7 | 0.023749348 |
| Irf9 | Irf7 | 0.029235918 |
| Irf9 | Stat3 | 0.021346051 |
| Irf9 | Irf1 | 0.012565434 |
| Ifit1 | Tmem173 | 0.13401803 |
| Ifit1 | Irf9 | 0.08346602 |
| Ccl2 | Tlr4 | 0.001291289 |
| Cd274 | Tlr3 | 0.001300067 |
| Cd274 | Tlr4 | 0.000773724 |
| Tap1 | Bst2 | 0.003033531 |
| Tap1 | Tlr3 | 0.001366193 |
| Tap1 | Tlr4 | 0.000562071 |
| Tap1 | Cd274 | 0.001841195 |
| Stat2 | Tlr3 | 0.002036073 |
| Stat2 | Tlr4 | 0.000906308 |
| Stat2 | Cd274 | 0.001362512 |
| Stat2 | Tap1 | 0.001691046 |
| Irgm1 | Tap1 | 0.00404264 |
| Irgm1 | Stat2 | 0.008182135 |
| Tlr2 | Bst2 | 0.002453153 |
| Tlr2 | Tlr3 | 0.001302157 |
| Tlr2 | Tlr4 | 0.00070282 |
| Tlr2 | Cd274 | 0.000759332 |
| Tlr2 | Tap1 | 0.001662925 |
| Tlr2 | Stat2 | 0.001083575 |
| Irf5 | Tlr3 | 0.002946498 |
| Irf5 | Tap1 | 0.001683796 |
| Irf5 | Tlr2 | 0.00143275 |
| Irf7 | Tlr3 | 0.001478097 |
| Irf7 | Tlr4 | 0.000614853 |
| Irf7 | Cd274 | 0.00134809 |
| Irf7 | Tap1 | 0.001738922 |
| Irf7 | Stat2 | 0.001573467 |
| Irf7 | Tlr2 | 0.001448857 |
| Irf7 | Irf5 | 0.001861336 |
| Ifnar1 | Tlr3 | 0.001174033 |
| Ifnar1 | Tlr4 | 0.001074882 |
| Ifnar1 | Ccl2 | 0.00238036 |
| Ifnar1 | Cd274 | 0.001241418 |
| Ifnar1 | Tap1 | 0.000782509 |
| Ifnar1 | Stat2 | 0.007707072 |
| Ifnar1 | Tlr2 | 0.000701941 |
| Ifnar1 | Irf7 | 0.001075123 |
| Ifi35 | Cd274 | 0.002376506 |
| Ifi35 | Tap1 | 0.00356505 |
| Ifi35 | Tlr2 | 0.002629225 |
| Relb | Ddx58 | 0.000632532 |
| Relb | Tlr3 | 0.000489163 |
| Relb | Tlr4 | 0.000466235 |
| Relb | Cd274 | 0.00065664 |
| Relb | Tap1 | 0.000521148 |
| Relb | Stat2 | 0.000726162 |
| Relb | Tlr2 | 0.000519005 |
| Relb | Irf7 | 0.000411938 |
| Relb | Ifnar1 | 0.000520342 |
| Stat3 | Ddx58 | 0.001014399 |
| Stat3 | Tlr3 | 0.00053042 |
| Stat3 | Tlr4 | 0.000510255 |
| Stat3 | Ccl2 | 0.001006132 |
| Stat3 | Cd274 | 0.000661674 |
| Stat3 | Tap1 | 0.000392561 |
| Stat3 | Stat2 | 0.000575371 |
| Stat3 | Tlr2 | 0.000770749 |
| Stat3 | Irf7 | 0.000386473 |
| Stat3 | Ifnar1 | 0.002474691 |
| Stat3 | Relb | 0.000226009 |
| Ccl5 | Tlr3 | 0.001265971 |
| Ccl5 | Tlr4 | 0.000648416 |
| Ccl5 | Cd274 | 0.001364387 |
| Ccl5 | Tap1 | 0.001740473 |
| Ccl5 | Stat2 | 0.001937855 |
| Ccl5 | Cd86 | 0.002174995 |
| Ccl5 | Irgm1 | 0.00450451 |
| Ccl5 | Tlr2 | 0.001305858 |
| Ccl5 | Irf7 | 0.001314969 |
| Ccl5 | Ifnar1 | 0.000924803 |
| Ccl5 | Ifi35 | 0.004117067 |
| Ccl5 | Relb | 0.000432224 |
| Ccl5 | Stat3 | 0.000425331 |
| Irf1 | Tlr3 | 0.001582176 |
| Irf1 | Tlr4 | 0.000774292 |
| Irf1 | Cd274 | 0.001770833 |
| Irf1 | Tap1 | 0.001509953 |
| Irf1 | Stat2 | 0.001441512 |
| Irf1 | Tlr2 | 0.001093413 |
| Irf1 | Irf7 | 0.001747055 |
| Irf1 | Ifnar1 | 0.000899222 |
| Irf1 | Ifi35 | 0.002476435 |
| Irf1 | Relb | 0.000491901 |
| Irf1 | Stat3 | 0.000461639 |
| Irf1 | Ccl5 | 0.001122595 |
| Tbk1 | Ddx58 | 0.001809052 |
| Tbk1 | Relb | 0.000542799 |
| Nmi | Tap1 | 0.00194281 |
| Nmi | Ifi35 | 0.033915147 |
| Nmi | Stat3 | 0.003193442 |
| Nmi | Ccl5 | 0.001724579 |
| Nmi | Irf1 | 0.001296498 |
| Nmi | Tbk1 | 0.001035431 |
| Stat1 | Ddx58 | 0.001783673 |
| Stat1 | Tlr3 | 0.000665232 |
| Stat1 | Tlr4 | 0.000261417 |
| Stat1 | Cd274 | 0.001185847 |
| Stat1 | Tap1 | 0.001261644 |
| Stat1 | Stat2 | 0.003107011 |
| Stat1 | Tlr2 | 0.001018253 |
| Stat1 | Irf7 | 0.001673138 |
| Stat1 | Ifnar1 | 0.000369792 |
| Stat1 | Ifi35 | 0.001690509 |
| Stat1 | Relb | 0.000508378 |
| Stat1 | Stat3 | 0.001189904 |
| Stat1 | Ccl5 | 0.000930691 |
| Stat1 | Irf1 | 0.00211282 |
| Stat1 | Tbk1 | 0.000341089 |
| Stat1 | Nmi | 0.004001989 |
| Irf9 | Tlr3 | 0.001893026 |
| Irf9 | Tlr4 | 0.000871881 |
| Irf9 | Cd274 | 0.00126539 |
| Irf9 | Tap1 | 0.002171746 |
| Irf9 | Stat2 | 0.013529695 |
| Irf9 | Irgm1 | 0.013688589 |
| Irf9 | Tlr2 | 0.00160138 |
| Irf9 | Irf7 | 0.002085762 |
| Irf9 | Ifnar1 | 0.001955182 |
| Irf9 | Relb | 0.000830994 |
| Irf9 | Stat3 | 0.000620234 |
| Irf9 | Ccl5 | 0.002052686 |
| Irf9 | Irf1 | 0.00165166 |
| Irf9 | Stat1 | 0.003925003 |
| Cxcl9 | Bst2 | 0.018284949 |
| Cxcl9 | Tap1 | 0.004654424 |
| Cxcl9 | Tlr2 | 0.004494559 |
| Cxcl9 | Ccl5 | 0.005632186 |
| Cxcl9 | Stat1 | 0.002365525 |
| Igtp | Tlr4 | 0.001457323 |
| Igtp | Ccl2 | 0.006339686 |
| Igtp | Cd274 | 0.001601399 |
| Igtp | Tap1 | 0.003066963 |
| Igtp | Stat2 | 0.0029517 |
| Igtp | Irgm1 | 0.013049886 |
| Igtp | Tlr2 | 0.001624569 |
| Igtp | Irf5 | 0.00476919 |
| Igtp | Irf7 | 0.002187683 |
| Igtp | Relb | 0.000930337 |
| Igtp | Ccl5 | 0.003462988 |
| Igtp | Irf1 | 0.001952011 |
| Igtp | Stat1 | 0.001741959 |
| Igtp | Irf9 | 0.006603433 |
| Usp18 | Tlr4 | 0.001399297 |
| Usp18 | Cd274 | 0.000778624 |
| Usp18 | Tap1 | 0.000655148 |
| Usp18 | Tlr2 | 0.001072318 |
| Usp18 | Relb | 0.000699944 |
| Usp18 | Tbk1 | 0.001252255 |
| Usp18 | Nmi | 0.001432482 |
| Usp18 | Stat1 | 0.000666675 |
| Rtp4 | Bst2 | 0.017055573 |
| Rtp4 | Tap1 | 0.004152132 |
| Rtp4 | Stat2 | 0.006776438 |
| Rtp4 | Tlr2 | 0.003290108 |
| Rtp4 | Irf7 | 0.00561502 |
| Rtp4 | Ccl5 | 0.003838907 |
| Ifit1 | Irf7 | 0.007366569 |
| Ifih1 | Tap1 | 0.002147602 |
| Ifih1 | Tlr2 | 0.001917346 |
| Ifih1 | Irf7 | 0.002406025 |
| Ifih1 | Ifi35 | 0.010416811 |
| Ifih1 | Irf1 | 0.003001997 |
| Ifih1 | Stat1 | 0.00123015 |
| Tapbp | Tap1 | 0.03602472 |
| Tapbp | Irf7 | 0.004236695 |
| Tapbp | Irf1 | 0.00437691 |
| Tapbp | Stat1 | 0.001878824 |
| Tapbp | Rtp4 | 0.02824194 |
| Tapbp | Ifih1 | 0.015055456 |
| Eif2ak2 | Bst2 | 0.004163093 |
| Eif2ak2 | Tlr3 | 0.001556737 |
| Eif2ak2 | Tlr4 | 0.001217539 |
| Eif2ak2 | Ccl2 | 0.003044451 |
| Eif2ak2 | Cd274 | 0.001164981 |
| Eif2ak2 | Tap1 | 0.000972306 |
| Eif2ak2 | Stat2 | 0.001617182 |
| Eif2ak2 | Cd86 | 0.002492108 |
| Eif2ak2 | Tlr2 | 0.001325693 |
| Eif2ak2 | Irf7 | 0.001145545 |
| Eif2ak2 | Ifnar1 | 0.000865524 |
| Eif2ak2 | Relb | 0.000483561 |
| Eif2ak2 | Stat3 | 0.002881588 |
| Eif2ak2 | Ccl5 | 0.000960866 |
| Eif2ak2 | Irf1 | 0.001229928 |
| Eif2ak2 | Nmi | 0.001465272 |
| Eif2ak2 | Stat1 | 0.002494557 |
| Eif2ak2 | Irf9 | 0.001897736 |
| Eif2ak2 | Igtp | 0.00249145 |
| Trim30a | Ddx58 | 0.011481475 |
| Trim30a | Tap1 | 0.004029927 |
| Trim30a | Ccl5 | 0.003892932 |
| Trim30a | Nmi | 0.007268284 |
| Parp9 | Tap1 | 0.002415453 |
| Parp9 | Irf7 | 0.003653692 |
| Parp9 | Ifi35 | 0.01072197 |
| Parp9 | Ccl5 | 0.002390838 |
| Parp9 | Irf1 | 0.003648058 |
| Parp9 | Stat1 | 0.001302858 |
| Parp9 | Ifih1 | 0.009813609 |
| Parp9 | Tapbp | 0.016846094 |
| Zbp1 | Bst2 | 0.073242225 |
| Tlr4 | Tlr3 | 0.005376491 |
| Cxcl10 | Ccl2 | 0.032753192 |
| Tlr2 | Tlr3 | 0.005122307 |
| Tlr2 | Tlr4 | 0.005216201 |
| Irf7 | Irf5 | 0.11565095 |
| Ifnar1 | Stat2 | 0.023557106 |
| Relb | Stat2 | 0.010706298 |
| Stat3 | Stat2 | 0.007459393 |
| Stat3 | Ifnar1 | 0.011009095 |
| Stat3 | Relb | 0.005533776 |
| Ccl5 | Ccl2 | 0.028380025 |
| Ccl5 | Cxcl10 | 0.02932831 |
| Irf1 | Irf5 | 0.08242843 |
| Irf1 | Irf7 | 0.09114199 |
| Tbk1 | Relb | 0.003148811 |
| Nmi | Ifi35 | 0.8292072 |
| Nmi | Stat3 | 0.02339972 |
| Stat1 | Stat2 | 0.008247389 |
| Stat1 | Relb | 0.007116241 |
| Stat1 | Stat3 | 0.004152199 |
| Stat1 | Irf1 | 0.012344101 |
| Stat1 | Nmi | 0.027977116 |
| Irf9 | Stat2 | 0.03362634 |
| Irf9 | Irf5 | 0.10191223 |
| Irf9 | Irf7 | 0.11069307 |
| Irf9 | Irf1 | 0.08212031 |
| Irf9 | Stat1 | 0.018828433 |
| Cxcl9 | Ccl2 | 0.031251628 |
| Cxcl9 | Cxcl10 | 0.034874525 |
| Cxcl9 | Ccl5 | 0.029888045 |
| Igtp | Irgm1 | 0.17088227 |
| Irgm2 | Irgm1 | 0.13903952 |
| Irgm2 | Igtp | 0.15280998 |
| Ifih1 | Ddx58 | 0.010435513 |
| Ifit2 | Ifit1 | 0.01484982 |
| Tapbp | Tap1 | 0.022319766 |
| Eif2ak2 | Stat3 | 0.002616776 |
| Eif2ak2 | Tbk1 | 0.002467275 |
| Eif2ak2 | Stat1 | 0.002817512 |
| Parp9 | Parp14 | 0.06813025 |
| Ifi47 | Irgm1 | 0.13300307 |
| Ifi47 | Igtp | 0.13102078 |
| Ifi47 | Irgm2 | 0.14388599 |
| Tbk1 | Irf7 | 0.14790572 |
